# Global dynamics of avian influenza: a twenty-year analysis of highly pathogenic viruses linking the Caspian Basin, Eurasia and Africa sectors (2005-2025)

**DOI:** 10.3389/fcimb.2026.1795327

**Published:** 2026-07-06

**Authors:** Alimurad Gadzhiev, Guy Petherbridge, Alexander Alekseev, Batyrgishi Mutashev, Kirill Sharshov, Ivan Sobolev, Madina Daudova, Maxim Perkovskii, Sasan Fereidouni, Alexander Shestopalov

**Affiliations:** 1 Institute of Ecology and Sustainable Development, Dagestan State University, Makhachkala, Russia; 2 Caspian Centre for Nature Conservation, Association of Universities and Research Centers of Caspian Region States, Makhachkala, Russia; 3 Research Institute of Virology, Federal Research Centre for Fundamental and Translational Medicine, Siberian Branch, Russian Academy of Sciences, Novosibirsk, Russia; 4 Laboratory of Ornithology, Astrakhan State Nature Biosphere Reserve, Astrakhan, Russia; 5 Research Institute of Wildlife Ecology, University of Veterinary Medicine, Vienna, Austria

**Keywords:** avian influenza, bidirectional viral movement, Caspian Basin-Eurasia-Africa, enzootic, epidemiological system, H5N1, panzootic, waterbirds

## Abstract

Highly pathogenic avian influenza (HPAI) viruses of HxNy subtypes have dispersed across continents since 2005 through the movements of wild aquatic birds. This study synthesizes virological and ecological evidence and graphic visualizations (2005–2025) as to how avian influenza viruses (AIV) in aquatic avifauna linking the Caspian Basin, Eurasia and Africa have been transmitted and evolved. The initial westward incursion of virulent H5N1 in 2005–2006 established the Caspian Sea region as a dynamic nexus for viral dispersal. First identified in East Asia and transmitted to Siberian breeding sites, the virus reached the Caspian, then rapidly spread to Europe and Africa via migratory waterbirds. In subsequent years, H5N1 became enzootic in poultry in Egypt and elsewhere in Africa. In parallel, low-pathogenic H9N2 also became established in poultry systems in Egypt and the Maghreb and later contributed a PA gene segment to reassortant H5N1 in West Africa. A decade later, the Caspian flyways again conveyed emergent HxNy strains: in winter 2016–2017, H5N8 (clade 2.3.4.4b) caused mass die-offs of swans, ducks and other waterbirds in Iran and neighboring countries. Since 2020, this lineage has swept across Eurasia, Africa and the Americas in a panzootic, with the Caspian–Black Sea region at the convergence of intercontinental transmission flyways. Eurasian-origin viruses were carried into Africa by southward-moving waterbirds, and in turn African-origin gene segments reappeared in H5N1 strains that were transmitted north to Caspian breeding colonies in 2022. Bidirectional viral movement also has occurred between Western Europe and northern Eurasia. The aquatic bird flyway zones linking Africa and Eurasia can therefore be seen as a single global epidemiological system or enzootic continuum. Knowledge of the virological dynamics outlined in this study is important for surveillance, early warning and response to highly pathogenic avian influenza in both animal and public health at a time when the incidence of infections and threats of more virulent reassortants are assessed as critically high.

## 1 Introduction

**Figure 1:**
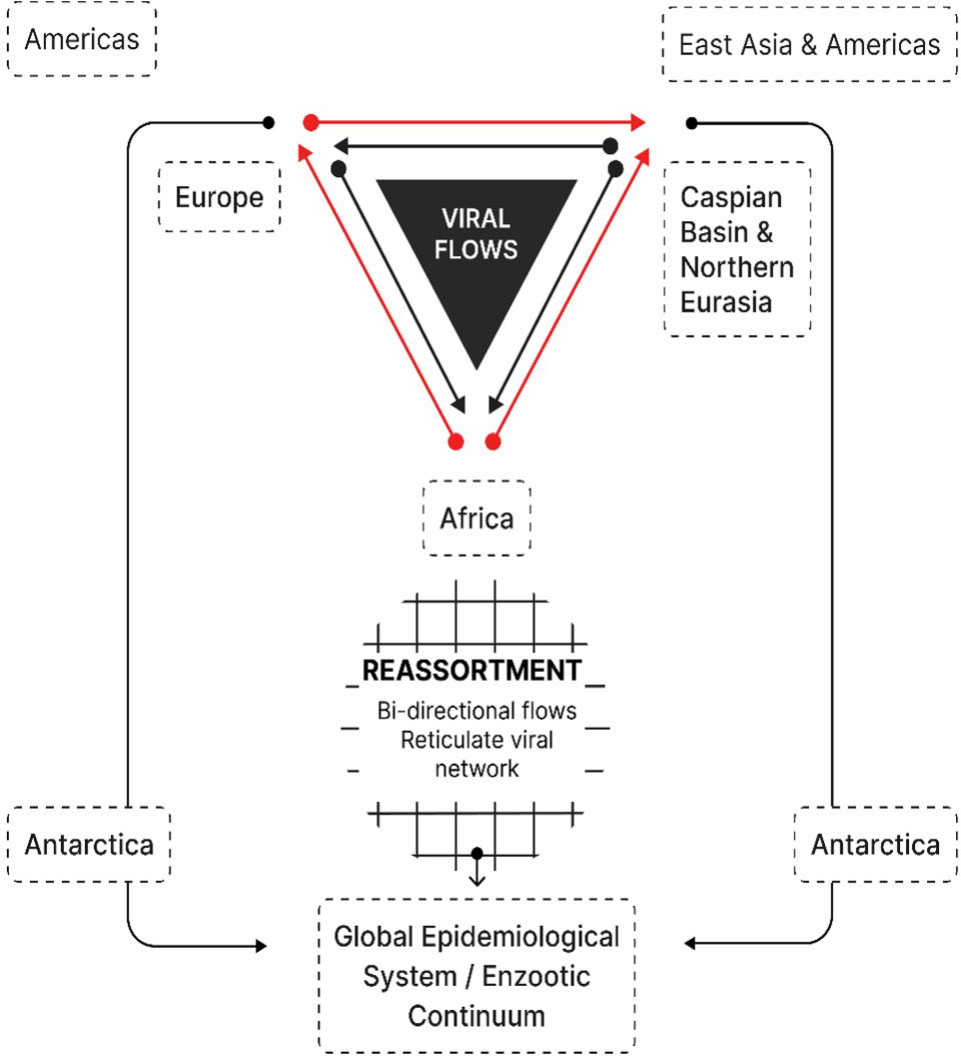
Conceptual flow chart of thesis of the present review.

In this narrative synthesis (**Figure 1**), we review two decades (2005–2025) of published genetic, epidemiological, and ecological evidence on highly pathogenic avian influenza H5Nx dynamics across three principal geographic connections: (a) the Caspian Basin and Africa, (b) Africa and Europe, and (c) Europe and the Caspian Basin, integrating both English-language and regional sources. We first outline the chronology and geography of major avian influenza epizootics, then examine evidence for bidirectional transmission and a broader interconnected epidemiological system, and finally consider environmental and surveillance-related factors that may influence these dynamics. We use the term “bidirectional” to refer to lineage movement in both directions - for example, from Eurasia to Africa during autumn migration, and potentially northward during spring migration.

Since 2005 a series of panzootic waves of highly pathogenic avian influenza viruses have spread westwards from East Asia across the Asian continent to Europe and Africa. All these major HxNy waves (Liu et al., 2005) have involved the Caspian Basin, either as a corridor or a crucible for new viral reassortants. Wild waterbirds – especially waterfowl (orders Anseriformes and Charadriiformes) – are natural reservoir hosts for these avian influenza A viruses (AIV). These birds typically carry low-pathogenic avian influenza (LPAI) strains asymptomatically, allowing viruses to circulate and be carried over long distances during migration (Guberti et al., 2007). 16 known hemagglutinin (H1–H16) and 9 neuraminidase (N1–N9) subtypes of AIV have been found in wild birds. Mild LPAI infections as well as the ability of AIV to persist in aquatic habitats for weeks or much longer under certain conditions enable viral reassortment into novel strains. This in turn may infect more vulnerable taxa. These factors set the stage for long-distance viral dispersal along the routes that birds travel between breeding and wintering grounds.

**Figure 2:**
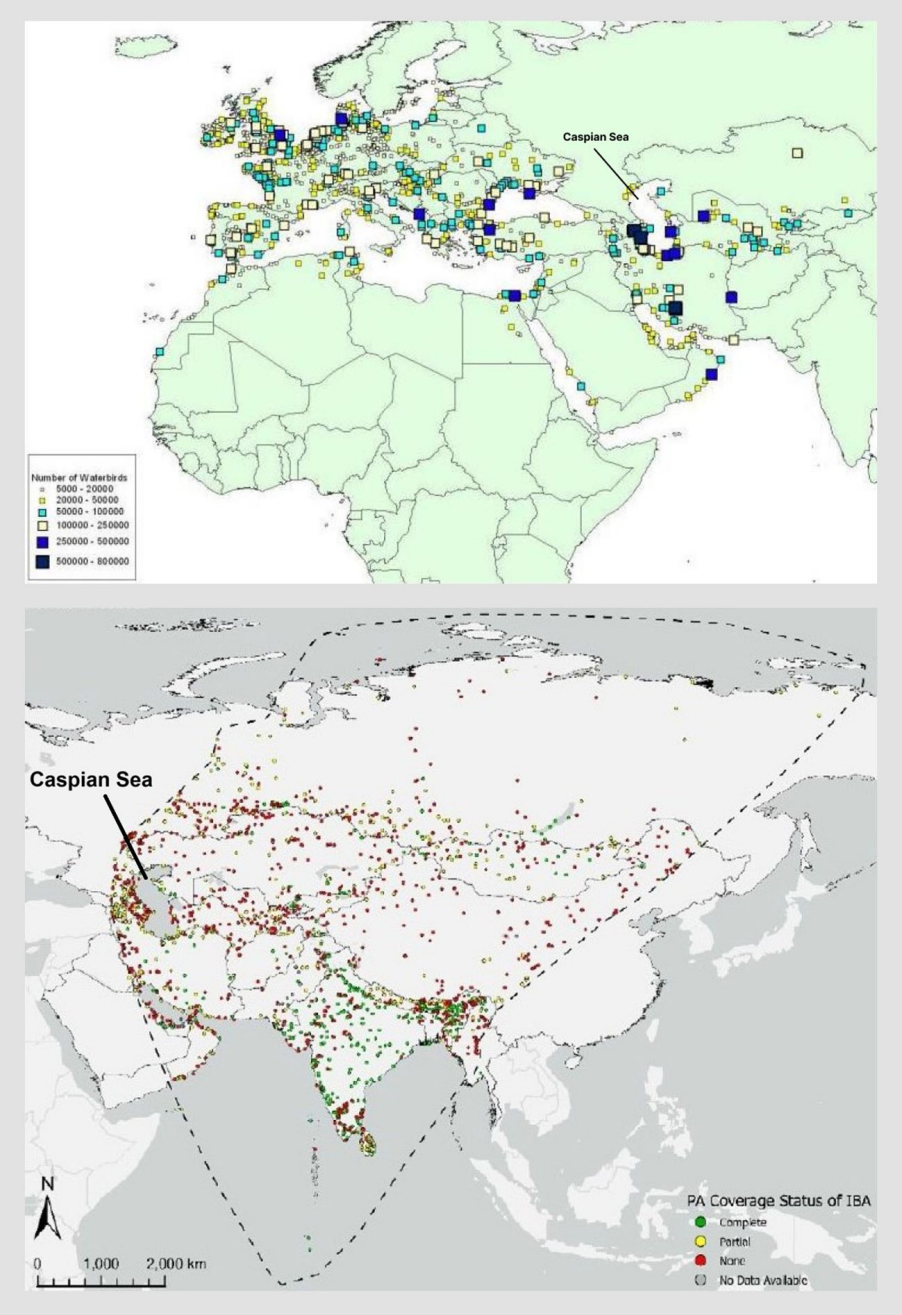
**(A)** Locations of waterbird species considered of high risk for highly pathogenic avian influenza in the Western Palaearctic and Southwest Asia. These are locations where combined counts of such species exceeded 20,000 between 1990 and 2005 and where two or more of these species occurred in numbers exceeding thresholds of 100, 250 or 500 depending on the species. European Commission & Delany et al., 2006. **(B)** Important Bird Areas (IBAs) in the zone of the Central Asia Flyway plotted by the Convention on Migratory Species provides a similar indication of the high concentration of bird species in the Caspian Basin as in **Figure 2a**. Birdlife International for Convention on Migratory Species, 2023.

In 2006, concerned by the particularly virulent spread of the H5N1 avian influenza virus in Europe (Wetlands International, 2002), the European Commission undertook an assessment of relevant ornithological and ecological data by Delany et al. (2006), which determined that the Caspian Basin has the largest resident or migratory populations in the Western Palaearctic and Southwest Asia of waterbird species considered of high risk for HPAI. (**Figure 2a**) These species are: Eurasian wigeon (*Mareca penelope*), Eurasian teal (*Anas crecca*), mallard (*Anas platyrhynchos*), northern pintail (*Anas acuta*), garganey (*Spatula querquedula*), northern shoveler (*Spatula clypeata*), marbled teal (*Marmaronetta angustirostris*), red crested pochard (*Netta rufina*), common pochard (*Aythya ferina*) and tufted duck (*Aythya fuligula*). The map of Important Bird Areas (IBAs) in the zone of the Central Asia Flyway produced by the Convention on Migratory Species provides a similar indication of the high concentration of bird species in the Caspian Basin (**Figure 2b**). The study presented here extends the tracking of the phenology of populations of certain of these species further south to the African continent.

Migration across this vast territory does not follow a single linear pathway. Rather, multiple partially overlapping routes connect Eurasian breeding areas with wintering regions in Europe and Africa. Along the western Caspian littoral, movements may be oriented both longitudinally and latitudinally, reflecting species-specific behavior and environmental conditions. As a result, different migratory flows can pass through the same region at the same time, linking otherwise distant ecological systems.

These patterns have led to the recognition that Afro-Palaearctic flyways may function not as isolated corridors, but as parts of a broader, interconnected migratory system. From a virological perspective, this implies that avian influenza circulation is shaped not only by long-distance dispersal, but also by repeated mixing, local persistence, and reassortment within key ecological nodes. In this context, the Caspian Basin can be viewed as one of the principal interfaces within a wider Africa–Eurasia avian influenza system.

### 1.1 Palaearctic-Afro-Tropical migratory flyways and connectivity between biogeographical regions

The biogeographical regions of interest here are interconnected by commonly recognized bird migration flyway zones, which delineate the major routes used by migratory species between breeding and wintering areas (Boere and Stroud, 2006; Galbraith et al., 2014).

**Figure 3:**
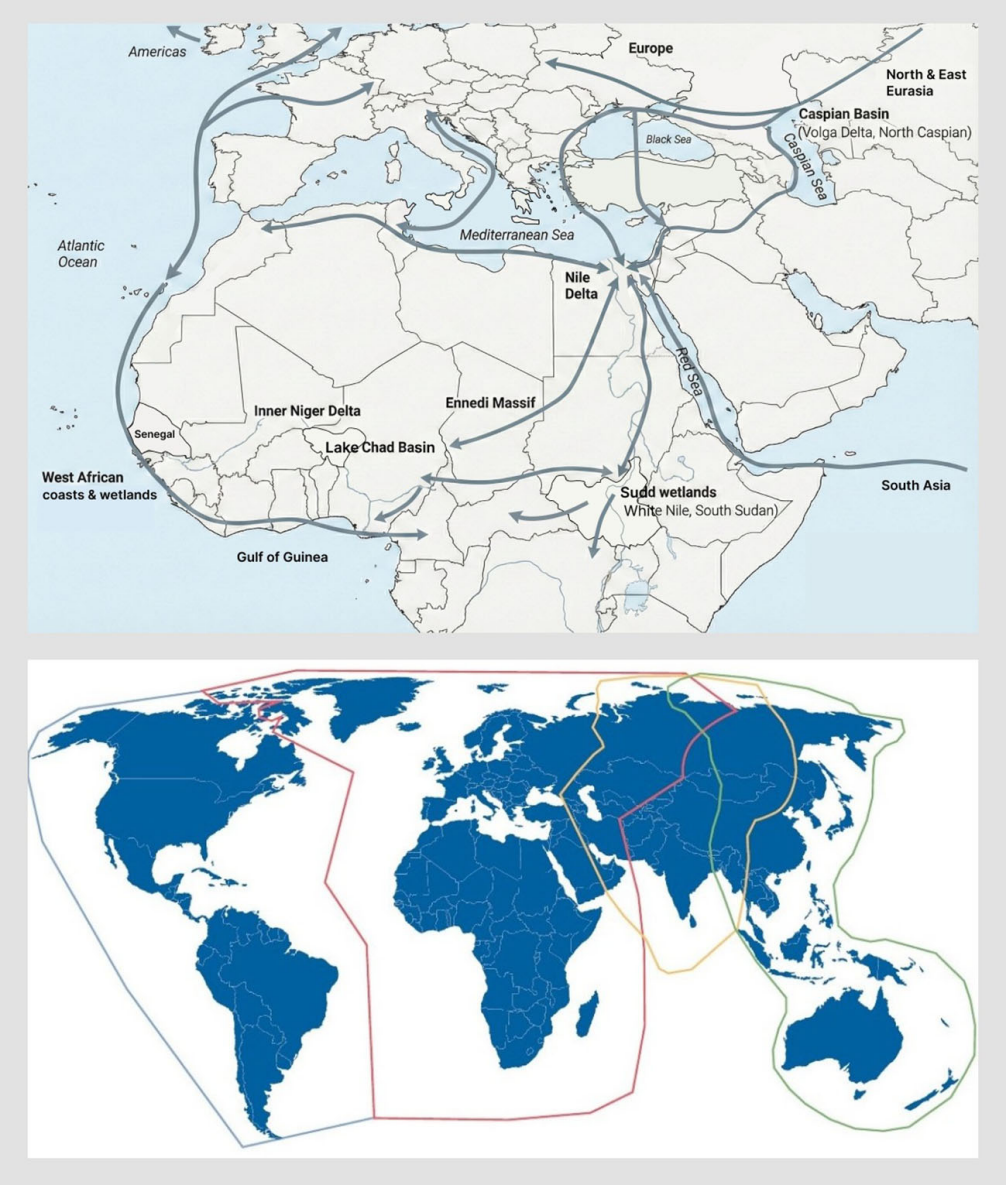
**(A)** Principal avian migratory corridors linking the Caspian Basin, Eurasia and Africa. **(B)** Global map aggregating accepted primary migratory bird zones. The Africa, Western Palaearctic and northeast America zone is that outlined in red (Galbraith et al., 2014).

**Figure 3a** is a schematic representation of the major Palaearctic–Afro-Tropical waterbird migration pathways structuring the Caspian–Eurasia–Africa avian influenza transmission system. These flyways follow the topography in skirting the barriers of most higher elevations, such as the Urals and the Caucasus massif. Another primary factor determining waterbird pathways is the presence of wetlands, lakes and rivers spaced at distances providing sustenance. The figure highlights the Caspian Basin as a central ecological hub of this system with bifurcation into a north-western corridor towards Europe to join the Black Sea–Mediterranean flyway and a southern corridor along the Caspian western coast towards Africa via north western Iran, Mesopotamia, Levant and the Nile delta. Secondary connections within Africa, including the Sudd wetlands, the Lake Chad basin, northeastern Nigeria, the West African coastal flyway and its extension into Central Africa. From the Sudd wetlands there is also another route southward to the Congo wetlands. This diagram is conceptual and intended to illustrate system-level connectivity rather than precise migratory tracks or temporal dynamics.

One notable feature of the Caspian basin as a key convergence point of Eurasian-African flyways is the geomorphological bottleneck formed between the north-eastern edge of the Great Caucasus massif and the Dagestan coast that funnels huge numbers of waterbirds during seasonal migrations. Numerous bird populations breed in the wetlands of northern Eurasia and the northern Caspian basin which migrate to wintering areas in Europe or Africa, and then return northward in spring. These repeated seasonal movements create substantial opportunities for the geographic spread of pathogens, including avian influenza viruses (Mine et al., 2019).

For some species and populations flyways are distinct ‘pathways’ linking discrete networks of key stopover sites, whereas for others, flyways are broader and more dispersed (Madsen et al., 2014). The Convention on the Conservation of Migratory Species has aggregated diverse overlapping flyways into a single unified Palaearctic-African-North Atlantic migratory zone, better reflecting the complex interweaving pathways associated with avian influenza gene flows (Mundkur et al., 2023; Moreau, 1972). (**Figure 3b**).

Below, we detail evidence of AIV lineage movements across regional dualities, drawing on data from major outbreak events and genetic connectivity revealed by surveillance and phylogenetic studies. While the dominant pattern for HPAI spread has been from Eurasia (including Europe and the Caspian Basin) (Iverson et al., 2011; Lycett et al., 2020; Tian et al., 2023; Yang et al., 2023), southwards to Africa (Kalonda et al., 2020; Zwarts et al., 2023) and ultimately westwards to North America (zu Dohna et al., 2009; Bevins et al., 2022: Günther et al., 2022; Alkie et al., 2023), there are also confirmed instances and plausible inferences of northward (Africa-Eurasia) transmission (Brown, 2010).

## 2 Southward spread of HXNY avian influenza viruses

Systematic, continent-scale evidence for migratory-bird-mediated transmission of avian influenza viruses (AIV) from Europe to Africa was limited during the early phases of HPAI emergence, despite recognition that low-pathogenic AIV (LPAIV) circulate widely in wild birds (Enserink, 2006; Abolnik et al., 2006; Kilpatrick et al., 2006; Balicer et al., 2007; Gauthier-Clerc et al., 2007; Krauss et al., 2007).

The earliest internationally recognized isolation of an AIV from wild birds was subtype H5N3 from a migratory common tern (*Sterna hirundo*) in South Africa (A/tern/S.A./61 [H5N3]) (Rowan, 1962; Becker, 1966; Lycett et al., 2019). Following the emergence of the HPAI H5N1 lineage A/Goose/Guangdong/1/96 in China (1996), repeated southward introductions into Africa via Northern Eurasia have been documented, largely coincident with autumn migration along Afro-Palaearctic flyways (Food and Agriculture Organisation (FAO) and Office International des Épizooties (OIE), 2006; Fusaro et al., 2019; Xu et al., 2016). For orientation, the major southward “waves” discussed below are summarized in **Table 1**.

**Table 1:** Illustrative examples of bidirectional lineage/segment exchange and reassortment along the Africa/Southwest Asia-Caspian corridor (sources cited in text).

Wave/timeframe	Subtype/clade	Key Eurasian events and inferred route	African detections/key notes (selected evidence)
First wave (2005–2008)	H5N1 clade 2.2 (Qinghai-like)	May–Jun 2005: mass die-off of bar-headed geese at Lake Qinghai (China). Jul 2005: first outbreaks in N Eurasia (Novosibirsk, Russia; 64 outbreaks in 2005–2006). Late 2005–early 2006: rapid spread across Western Europe and SW Asia (**Figure 4**). Multiple introductions inferred from Eastern Europe/Black Sea–Caspian/Volga corridors; timing consistent with autumn migration from stopovers to West African wetlands.	Feb 2006: first tropical Africa HPAI detection in Nigerian poultry; viruses closely matched Black Sea wild bird strains from weeks earlier (Meseko et al., 2018). 2006 detections also in Sudan, Burkina Faso, Côte d’Ivoire, Cameroon (clade 2.2). ≥5 separate Eurasia→Africa introductions inferred: four lineages in Nigeria/Niger/Burkina Faso + one into Egypt (Owoade et al., 2008; Henning et al., 2012; Fusaro et al., 2019). Clade persisted ~9 years in West Africa, while Egypt’s poultry-endemic clade 2.2.1 persisted but remained largely contained (Arafa et al., 2016; Meseko et al., 2018; Kim, 2018).
Second wave (2014–2015)	H5N1 clade 2.3.2.1c	New introductions closely related to contemporaneous Asian strains; at least two near-simultaneous introductions inferred in late 2014 (Bi et al., 2016; Fusaro et al., 2019). North Caspian staging areas implicated by genomic similarity of a 2015 Kazakhstan flamingo isolate.	2014–2015 outbreaks in Côte d’Ivoire, Ghana, Niger, Nigeria, Burkina Faso (Fasanmi et al., 2017; Shittu et al., 2017; Suu-Ire et al., 2019; Verhagen et al., 2021). A/flamingo/Mangistau/6570/2015 (H5N1) (Kazakhstan) showed high similarity across all eight genes to West African poultry outbreak viruses; HA clustered within clade 2.3.2.1c circulating since 2014 in Eurasia/SW Asia/Eastern Europe/West Africa (Karamendin et al., 2020).
Third wave (2016–2017)	H5N8 clade 2.3.4.4b (group B)	Late 2016–2017: reassortant H5N8 expanded from China and the Russian Federation to Asia, SW Asia, Europe and western Africa (Global Consortium for H5N8 and Related Influenza Viruses, 2016). Multiple introductions into Africa inferred: (i) Eastern Europe → Egypt (via Nile Delta migrants) and (ii) north-central Asia (Russia/Mongolia) → Africa via Central Asia–SW Asia–Africa flyway, with the Caspian Basin as a key corridor; Siberian breeders may reach West Africa via Black Sea/Mediterranean routes (Najdenski et al., 2018).	By early 2017, detections across Africa (Egypt, Uganda, Nigeria, South Africa, and others), including first reports in central, eastern and southern Africa (Oluwayelu et al., 2017; Ameji et al., 2017; Selim et al., 2017; Twabela et al., 2018; Khomenko et al., 2018; Naguib et al., 2019; Chieloka, 2021).
Fourth wave (2020–2023)	H5N1 clade 2.3.4.4b	Late 2020: novel European H5N1 emerged and persisted year-round (Cui et al., 2022). Rapid Europe→West Africa movement documented by Jan 2021 (Pohlmann et al., 2022), followed by wider dissemination during subsequent southbound movements. Long-distance spread also reached the Americas and Antarctica (Gadzhiev et al., 2024).	By winter 2021–2022, outbreaks confirmed in multiple African countries (Ghana, Nigeria, Côte d’Ivoire, The Gambia; Egypt, Tunisia, Libya), affecting poultry and wild birds. Network analyzes show strong asymmetry: 18/22 inferred H5N1 transmission pathways were southward vs 4/22 northward (Xu et al., 2016).

### 2.1 Eurasia to Africa H5N1 clade 2.2 southward spread (first wave 2005)

In May–June 2005, HPAI H5N1 clade 2.2 caused a mass die-off of bar-headed geese at Lake Qinghai (China), representing the first widely documented large-scale H5N1 outbreak in migratory waterfowl and a precursor to intercontinental spread. Phylogenetic trees available prior to early 2005 (Liu et al., 2005) suggested that Qinghai viruses were primarily of local, East Asian or Australasian origin; notably, migrants from Australia and New Zealand congregate at Qinghai. Later in 2005, however, a highly virulent Qinghai-like strain appeared in northern Eurasia and further west (Liu et al., 2005).

**Figure 4:**
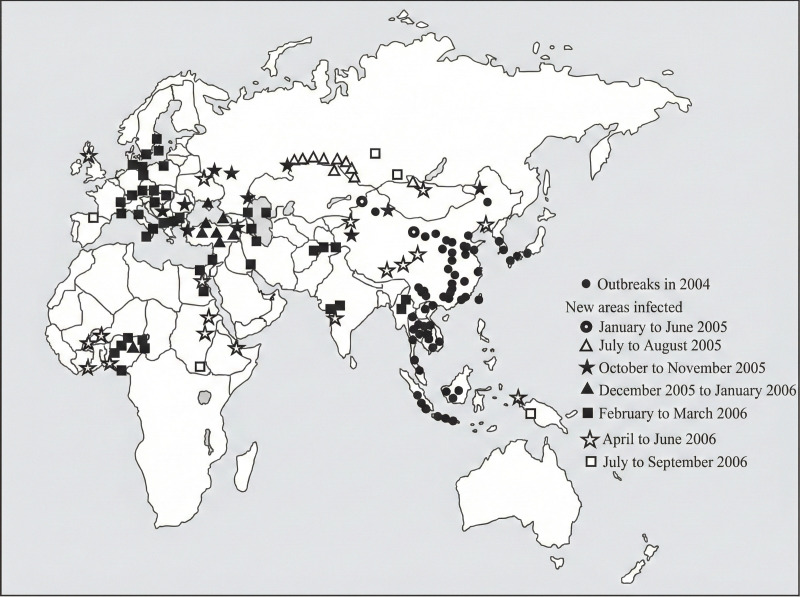
Global H5N1 outbreaks in 2004 and newly affected locations in 2005–2006. The map is based on disease situation reports provided by the World Health Organization, the Food and Agriculture Organization of the United Nations, and the World Organisation for Animal Health (WOAH/OIE). Williams and Peterson (2009).

By late 2005 and early 2006, outbreaks were reported in wild birds and poultry across Western Europe and southwest Asia (Williams and Peterson, 2009). (**Figure 4**) Genetic analyzes linked these viruses to a rapidly dispersing Qinghai-like clade 2.2 lineage with a common origin (Shestopalov et al., 2016; Bi et al., 2015). The first reported outbreaks in northern Eurasia occurred at the end of July 2005 in domestic poultry in Russia’s Novosibirsk Region (Lipatov et al., 2007); HA and NA genes were closely related to Asian outbreak viruses (2003–2005) and Japan (2003–2004), and Novosibirsk recorded 64 outbreaks in 2005–2006 affecting both wild and domestic birds (Shestopalov et al., 2016).

Phylogenetic reconstructions also hinted that movements might not be strictly one-way. A 2006 phylogeny of Lake Qinghai isolates showed re-emergence of viruses previously recorded in the region, consistent with (though not proving) return movements via migratory birds from other parts of Eurasia and Africa. However, Wang et al. (2008) did not exclude local circulation as an alternative explanation. In Germany (2006), phylogenetic trees compiled by Starick et al. (2008) included African isolates from Egypt, Nigeria, Sudan and Côte d’Ivoire, reinforcing the emerging picture of Afro-Palaearctic connectivity for clade 2.2.

#### 2.1.1 Sahel wetlands and seasonal climatic regime

The greatest concentration of wetlands in Africa lies between ~15°N and 20°N in the Sahel, including the Senegal River Basin (Senegal/Mauritania), the Niger River Delta/Basin (Mali) (Zwarts et al., 2009; Zwarts and Diallo, 2002), Lake Chad and the Logone/Chari system (Cameroon/Nigeria/Chad) (Mullié et al., 1999; Oyebande et al., 2003; Nwankwoala, 2012), and—further east—the Sudd wetlands on the White Nile (South Sudan) (Kabii, 1997). These wetlands provide key wintering habitats for Palaearctic migratory waterbirds (Turpie, 1996; Dodman et al., 1997; Girard and Thal, 2001; Fishpool and Evans, 2001).

**Figure 5:**
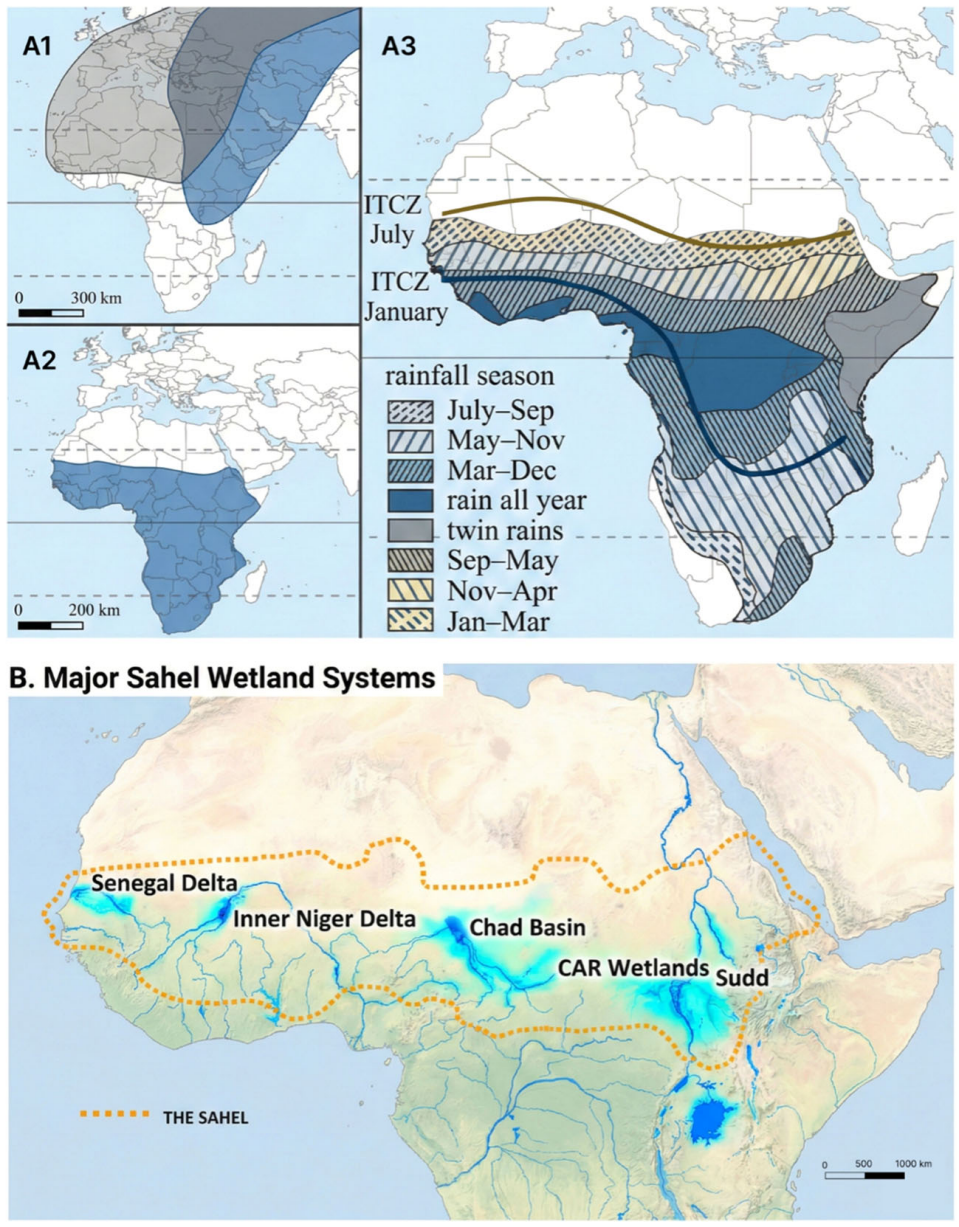
**(A)** Geographical position of the Inter-Tropical Convergence Zone **(ITCZ)** in sub-Saharan Africa in July and January: (A1) main migratory flyways and distribution range of Eurasian waterbirds, (A2) Afro-Tropical waterbirds in Sub-Saharan Africa and (A3) timing of the wet season and seasonal position of the Inter-Tropical Convergence Zone (ITCZ). Wilson and Primack, 2019. **(B)** Major Sahel wetland and river systems. Gaidet et al., 2012.

The Sahel’s climate is structured primarily latitudinally by rainfall rather than temperature, with a wet season (June to September/October) and a dry season (November to May). September and May are transition months; in this framing, September corresponds to the region’s “spring” and May to its “autumn” (Liebmann et al., 2012; Froidurot and Diedhiou, 2017). Seasonal wetland water availability tracks the Inter-Tropical Convergence Zone (ITCZ), which shifts northward in July and southward by January (Wilson and Primack, 2019) (**Figure 5a**). Gaidet et al. (2012) showed that, between July and January, European migrant waterbirds tend to occupy the seasonal position of the ITCZ.

Extensive West African surveillance detected LPAIV in both Palaearctic migrants and Afro-Tropical residents, demonstrating wintertime AIV circulation in sub-Saharan wetlands (Gaidet et al., 2007a, 2015; Snoeck et al., 2011). Detection of LPAIV—including H5 and other subtypes—in Eurasian ducks at major overwintering sites (Lake Chad Basin, Inner Niger Delta, Senegal River Delta; **Figure 5b**) supports the hypothesis that viruses can persist in wild duck populations through continuous circulation. Multiple subtypes isolated from garganey within a single wintering population (Inner Niger Delta, Mali) mirror the subtype diversity documented in Europe and North America and indicate simultaneous co-circulation of different lineages (Gaidet et al., 2007b, 2008).

From a conservation and disease-ecology perspective, Sahelian waterbird populations are increasingly stressed by irregular wet seasons, habitat destruction, and net-trapping for local consumption and markets. The Lake Chad Basin has contracted dramatically since 1963 due to water extraction and climate change—often compared to the Aral Sea shrinkage in Central Asia (Pham-Duc et al., 2020; Liebmann et al., 2012; Nagy et al., 2021). Such habitat transformation can alter waterbird density, mixing patterns and physiological stress, potentially affecting avian viral susceptibility and transmission dynamics (Cappelle et al., 2012; Vandegrift et al., 2010; Curseu et al., 2009; Hall, 2021; Alexander et al., 2024).

### 2.2 Eurasia to Africa H5N1 clade 2.2 southward spread (first wave 2006–2008)

Clade 2.2 reached tropical Africa by February 2006, when HPAI H5N1 was confirmed in Nigerian poultry—the first detection of HPAI in tropical Africa. Nigerian viruses were virtually identical to H5N1 detected in wild swans and geese in the Black Sea region weeks earlier, consistent with introduction via infected migratory waterbirds arriving at Nigerian wetlands (Meseko et al., 2018). Contemporary detections in Sudan, Burkina Faso, Côte d’Ivoire and Cameroon (Njouom et al., 2008; Kouam et al., 2019) were likewise traced to clade 2.2, indicating southbound dispersal from Eurasia into Africa during the 2005–2006 migration season.

**Figure 6:**
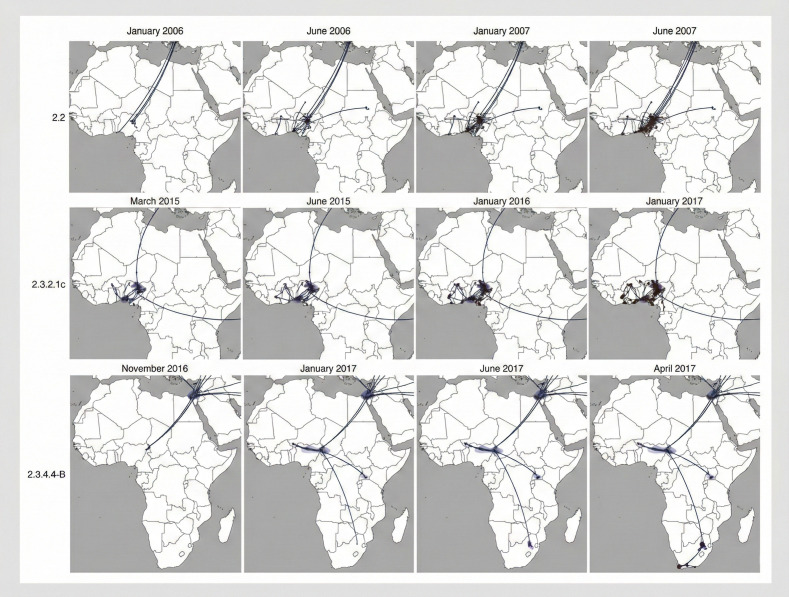
Africa: spatiotemporal dispersal of the three HPAI H5Nx clades. Dispersal patterns inferred using continuous phylogeographic analysis are shown for four time slices for each of the three H5Nx clades. Note the distinct change of the principal migration route to and from Africa: A(H5N1) clade 2.2. and clade 2.3.2.1c (directly from Europe – 2006 to 2007 & 2015-2017) and clade 2.3.4.4b (from the Caspian Basin via Egypt – 2016-2017). Fusaro et al., 2019. Note: for Nov.2016 – Apr. 2017 the maps do not show the important waterbird flyway to West Africa via the Sudd wetlands in South Sudan which continues to be used today (see **Figures 10**, **11a–d** below & discussion 2.3 Eurasia to Africa H5N1 clade).

Africa did not receive a single “founder” virus. Fusaro et al. (2019) identified three distinct clade 2.2 lineages circulating in Nigeria, Niger and Burkina Faso, plus an additional lineage introduced into Egypt, implying at least five separate Eurasia→Africa introduction events during this first wave (World Health Organisation (WHO), 2006; Owoade et al., 2008; Henning et al., 2012) (**Figure 6**). African isolates were most closely related to viruses circulating in Eastern Europe, including the Black Sea region and the Caspian Basin/Volga catchment (Fusaro et al., 2019). The estimated timing of introductions (late 2005 to early 2006) aligns with autumn migration, when ducks and shorebirds depart Black Sea/Caspian stopovers and reach West African wetlands by winter.

After 2006, clade 2.2 continued to be detected mainly in poultry. It became endemic in Egypt (Meleigy, 2007; Saad et al., 2007; Williams et al., 2008) and persisted in West Africa for approximately nine years before receding (Meseko et al., 2018). Surveillance in West Africa during 2006 did not detect H5N1 in healthy wild birds. Additional incursions occurred, including a new Eurasian introduction into Nigerian poultry in late 2007–2008. Egypt’s poultry-endemic clade 2.2.1 diverged but remained largely geographically contained within Egypt, with only sporadic spread to neighboring southwest Asia (Arafa et al., 2016; Kim, 2018; El-Shesheny et al., 2014).

### 2.3 Southward spread (second wave 2014–2015)

After several years during which African H5 infections largely involved clade 2.2, a second southward wave occurred in 2014–2015 with the introduction of HPAI H5N1 clade 2.3.2.1c. Outbreaks were reported across West Africa (Côte d’Ivoire, Ghana, Niger, Nigeria and Burkina Faso) (Tassoni et al., 2016; Fasanmi et al., 2017; Shittu et al., 2017; Suu-Ire et al., 2019; Verhagen et al., 2021). Phylogenetic analyzes indicated these viruses were not descendants of earlier African clade 2.2 lineages but represented new introductions closely related to contemporaneous Asian strains (Bi et al., 2016), with at least two nearly simultaneous introductions inferred in late 2014 (Fusaro et al., 2019).

A potential link between Eurasian staging areas and West African outbreaks was highlighted by genomic data from a dead greater flamingo sampled in 2015 in the Kazakh sector of the North Caspian Sea (A/flamingo/Mangistau/6570/2015 [H5N1]). BLAST analysis showed high similarity across all eight gene segments to H5N1 viruses isolated from poultry in Burkina Faso, Côte d’Ivoire, Ghana, Niger and Nigeria, and the HA gene clustered within clade 2.3.2.1c viruses detected since 2014 across Eurasia, southwest Asia, Eastern Europe and West Africa (Karamendin et al., 2020). Greater flamingos have a non-breeding range in the Sahel from Mauritania to Guinea-Bissau and Nigeria (Johnson, 1989), supporting the proposition that flamingos can contribute to intercontinental transfer and bidirectional movement of H5N1 variants (Karamendin et al., 2020).

### 2.4 Eurasia to Africa H5N8 clade 2.3.4.4b southward spread (third wave 2016–2017)

In late 2016, a reassortant HPAI H5N8 virus (clade 2.3.4.4, group B) expanded from China and the Russian Federation into Asia, southwest Asia, Europe and western Africa (Global Consortium for H5N8 and Related Influenza Viruses, 2016; Lee et al., 2017; Poen et al., 2018). The 2016–2017 season was marked by extensive wild-bird mortality and poultry outbreaks across Europe (Poen et al., 2018). During the same period, H5N8 was detected in domestic and wild birds across Africa from Egypt to Uganda, Nigeria and South Africa, including first reports in central, eastern and southern Africa (Oluwayelu et al., 2020; Ameji et al., 2017; Selim et al., 2017; Twabela et al., 2018; Khomenko et al., 2018; Naguib et al., 2019; Chieloka, 2021).

Phylogeographic analyzes have indicated multiple independent introductions into Africa. At least one introduction into North Africa (Egypt) likely originated from Eastern Europe via autumn migrants reaching the Nile Delta (Ibrahim, 2011). Other African H5N8 strains appeared to originate directly from north-central Asian viruses (Russia/Mongolia), implying that some migratory populations bypassed Europe and carried virus from Asian breeding areas to Africa along the Central Asia–Southwest Asia–Africa flyway, with the Caspian Basin acting as a principal corridor. This is consistent with the migration ecology of several Siberian breeders that reach West Africa via the Black Sea/Mediterranean corridor; infection along this route could seed outbreaks after arrival in African wetlands (Najdenski et al., 2018). By early 2017, H5N8 was widespread in African poultry and wild birds.

### 2.5 Eurasia to Africa H5N1 clade 2.3.4.4b southward spread (fourth wave 2020–2023)

The unprecedented H5N1 clade 2.3.4.4b epizootic of 2020–2023 continued and amplified this southward pattern (Cui et al., 2022). A novel H5N1 strain emerged in Europe in late 2020 and, unlike earlier largely winter-limited outbreaks, persisted year-round (King et al., 2021; Piesche et al., 2024). Closely related viruses were detected in West Africa by January 2021 (Pohlmann et al., 2022), and migratory birds departing Europe during subsequent southbound movements (notably autumn 2021) likely contributed to wider dissemination into Africa. By winter 2021–2022, outbreaks were confirmed in multiple African countries—Ghana, Nigeria, Côte d’Ivoire and Gambia in West Africa, and Egypt, Tunisia and Libya in North Africa—affecting both poultry and wild birds. In parallel, long-distance bird-mediated movements carried this clade to the Americas and even Antarctica (Gadzhiev et al., 2024).

Overall, phylogeographic reconstructions consistently identify autumn southbound migration as the dominant mechanism of Eurasia-to-Africa H5N1 gene flow (CMS FAO Co-convened Scientific Task Force on Avian Influenza and Wild Birds, 2022; BirdLife International, 2024). Network analysis of 22 inferred H5N1 transmission pathways found 18 southward versus four northward movements (Xu et al., 2016; Abolnik, 2010; Cumming et al., 2011), underscoring the asymmetry in intercontinental spread.

## 3 HXNY avian influenza viruses in Africa and northward spread to Europe

Although Africa is often downstream of Eurasian HPAI epicenters during autumn migration, multiple datasets indicate that AIV can circulate and reassort within African systems and that viral lineages or gene segments with African ancestry can move northward when migrants return to Eurasia in spring. Documented examples and the ecological context for such northward movements are synthesized below.

### 3.1 Africa to Western Europe northward spread of H5Nx strains (2019–2021)

A well-documented example of African ancestry contributing to European HPAI was reported by Świętoń et al. (2020), who described an HPAI H5N8 clade 2.3.4.4b virus in Europe generated by reassortment between an H5N8 subtype virus from sub-Saharan Africa and LPAI viruses from Eurasia.

Frequent reassortment and lineage diversification are key drivers of avian influenza virus evolution across Eurasian host systems (Meng et al., 2019).

In July 2019, surveillance in Nigerian live-bird markets detected HPAI H5N8 in guinea fowl in Ogun State. In late December 2019, Poland recorded suspected HPAI in 14-week-old meat turkeys near the fish ponds and lakes of the Łęczna–Włodawa Lakeland, with abrupt mortality (3,000–5,000 birds) during the first three days after onset. Laboratory characterization identified a novel European HPAI H5N8 genotype (clade 2.3.4.4b) comprising six gene segments from sub-Saharan African HPAI H5N8 viruses and two segments from Eurasian LPAI viruses (likely introduced through the Caspian Basin/Volga catchment). The Nigerian PB1 and NP genes grouped with 2018 viruses from South Africa; by contrast, the Polish virus clustered (for its Eurasian LPAI-derived segments) with viruses detected in recent years in northern Eurasian Lake Chany and the Kurgan region of Russia—known staging areas for migratory birds moving into Europe. The location of the reassortment event could not be resolved.

Świętoń et al. (2020) suggested that such Africa-to-Eurasia introductions could be facilitated by subclinical infections, insufficient active surveillance in healthy wild populations, expansion of HPAI into previously unaffected areas, and exposure changes linked to unusual weather-driven shifts in migration routes. The detection of closely related strains one year apart in two countries >5,000 km apart underscores both the mobility of HPAI and the likelihood of surveillance gaps.

Broader analyzes also support bidirectional exchange across Eurasia. Zhang et al. (2023) integrated phylogenetics and host ecology to report two-way gene flow of H5N8 between Europe and Asia, arguing that Europe cannot be treated solely as a sink: lineages sampled in Asia in late 2020 resembled viruses present earlier in Europe, and the Netherlands 2016–2017 event provided an example of local amplification and subsequent outward movement. Importantly, such long-distance phylogenetic links do not imply that a single bird transports virus from East Asia to Europe in one step; rather, under-sampled breeding or stopover regions (e.g., northern Asia) and “relay” transmission via partially migratory populations can generate apparent intercontinental jumps when intermediate data are missing.

Pohlmann et al. (2022) provide quantitative evidence that the recent HPAI H5N1 clade 2.3.4.4b wave in Western Europe is no longer adequately described as a winter-limited epizootic, but shows features consistent with persistent enzootic circulation, including sustained transmission beyond the traditional seasonal window and repeated re-emergence. Their phylogeographic reconstruction links Western Europe and West Africa within a rapidly cycling system, characterized by Europe-to-Africa spread followed by ongoing exchange and local evolution.

The chronology, phylogenetic structure, genetic similarity values, host breadth, and inferred pathway/residence-time metrics reported by Pohlmann et al. and used in this review are consolidated in **Table 2** (including evidence for West African reassortment and the emergence of distinct regional genetic groups).

**Table 2:** Key quantitative outputs from Pohlmann et al. (2022), summarizing (i) chronology of emergence and intercontinental detections, (ii) phylogenetic structure and lineage assignments, (iii) genetic similarity metrics, (iv) affected host breadth, (v) inferred global spread pathways and residence-time estimates, and (vi) evidence for reassortment and formation of regional genetic groups.

Section 3.1. quantitative outputs	Specific details
Core evidence in Section 3.1	Evidence for a transition from seasonal epizootics to persistent enzootic circulation; year-round H5N1 circulation in Western Europe and clear chronological linkage between European and West African strains.
Index emergence event	H5N1 clade 2.3.4.4b first emerged in Europe in October 2020 in the Netherlands via reassortment between H5N8 and low-pathogenic avian influenza viruses.
Persistence pattern	Unprecedented maintenance of highly pathogenic strains through the European summer months (all-year-round circulation in Western Europe).
Europe -> West Africa -> North America timeline	Europe to West Africa by January 2021; onward to North America in late 2021 via Atlantic pathways. The Europe-to-West Africa jump occurred within three months.
Genetic similarity	West African viruses showed 98-99% nucleotide identity to contemporary European strains; also 98-99% nucleotide identity to concurrent European and Middle Eastern isolates.
Phylogenetic structure	Two primary European branches: “East Asia” and “Eurasia/Africa”. West African strains consistently cluster within the Eurasia/Africa lineage.
2021 diversification and bidirectionality	Summer 2021: two European sublineages (B1 and B2). B1 shows African ancestral relationships in hemagglutinin genes. October 2021: B1 re-emerged in Europe, first detected in Eurasian wigeon and Eurasian teal in the Wadden Sea; December 2021: major West African outbreaks in Burkina Faso, Nigeria and Niger.
Host range/ecological impact	62 wild bird species affected, including 17 waterfowl species and 12 raptor species.
Global spread reconstruction	22 distinct spread pathways identified globally for clade 2.3.4.4b.
West African reassortment (WA1/WA2)	West African viruses reassorted locally with endemic H9N2 viruses, incorporating PA gene segments from the zoonotic G1 lineage circulating regionally during 2017-2020, yielding distinct WA1 and WA2 genetic groups.
Significance and timing constraints	Described as the most significant shift since emergence of virulent H5N1 in 1996. Intercontinental transmission showed lag times under six months; initial Europe-to-West Africa introduction occurred within three months, followed by synchronized outbreak patterns and regional diversification through local reassortment.

Collectively, these results support the interpretation that Africa is not merely a terminal sink for Eurasian HPAI incursions. Rather, sustained circulation and reassortment in Africa can contribute to subsequent Eurasian outbreak dynamics, consistent with the broader hypothesis. advanced here of an Afro-Eurasian enzootic continuum connected by overlapping flyways.

It should be noted here that monitoring capabilities and intensity vary substantially among regions, with comparatively dense surveillance networks in Europe and parts of Asia, but more limited and discontinuous coverage in many African regions and parts of northern Eurasia. Consequently, some inferred residence times or lineage transitions may partially reflect differences in sampling intensity rather than true differences in viral circulation dynamics.

Notably, this West African reassortment signal is consistent with the broader African H9N2 literature: H9N2 has circulated in poultry in Egypt and across the Maghreb, has undergone local genetic diversification, and by 2021 had contributed a PA gene segment to reassortant clade 2.3.4.4b H5N1 in Burkina Faso (Nagy et al., 2017; Tombari et al., 2011; Gomaa et al., 2015; Aouini et al., 2016; El-Houadfi et al., 2016; El-Houadfi et al., 2025; Arbi et al., 2020; Barberis et al., 2020; Larbi et al., 2024; Ouoba et al., 2022).

Evidence for multi-flyway gene flow is also apparent in African LPAIV datasets. In Egypt (2003–2007), Gerloff et al. (2013) sequenced 28 LPAIV genomes (15 subtypes) primarily from migratory waterfowl in the Nile Delta and found internal genes derived from viruses associated with up to four different flyways; molecular clock analysis suggested the most recent common ancestors existed 5–10 years before sampling, implying frequent reassortment and exchange. Mine et al. (2019) similarly reported N6 neuraminidase genes from African and European strains clustering in a shared clade, including a strain from a great white pelican associated with a population migrating between Europe and Africa via Israel, where the Black Sea–Mediterranean and West Asian–East African flyways overlap.

### 3.2 African environment: dynamics of transmission, circulation, and potential reassortment

Surveillance in early 2006 across 12 sub-Saharan countries detected AIV exclusively of low pathogenicity, with an overall prevalence of 3.5% in sampled wild birds, including both Eurasian migratory ducks and African resident ducks (Gaidet et al., 2007a). Isolates included H1N1, H5N3, H11N9 and H12N5 from healthy migratory garganey and resident white-faced whistling ducks (*Dendrocygna viduata*). Garganey are the most abundant Eurasia-Africa migrant duck (~2 million wintering in West African wetlands) (Gaidet et al., 2012). No HPAI H5N1 was detected in healthy wild birds during these surveys, suggesting that early African HPAI activity was largely poultry-associated; nevertheless, the presence of LPAIV H5 and other subtypes implies that infected migrants could potentially transport viruses back north if infections occur near the end of the wintering period.

A continent-wide analysis linked AIV prevalence in African wildfowl primarily to the influx of Palaearctic migrants and the seasonal ecology of Sahelian wetlands rather than to local factors (Gaidet et al., 2012). In the Sahel, prevalence peaks after the rainy season as shrinking wetlands concentrate waterfowl and coincide with peak migrant abundance; for example, Eurasian ducks join resident ducks in large mixed flocks during January–February, then disperse as wetlands contract further. These dynamics can facilitate both within-Africa spread (via resident dispersal) and northward export (via spring departure of Palaearctic migrants).

In the Inner Niger Delta (Mali), AIV was detected in all seasons, albeit at very low prevalence during the hot dry season, consistent with year-round circulation maintained at low levels in resident waterfowl and replenished each winter by migrants (Cappelle et al., 2012). These patterns support the hypothesis that West Africa can function as both a maintenance and reassortment zone, particularly where multiple lineages co-circulate (Pandit et al., 2013; Xu et al., 2016).

Egypt experienced a different dynamic: clade 2.2.1 became entrenched in poultry and diverged, raising concerns that migratory birds mixing in Egyptian wetlands could acquire endemic strains. In practice, however, this poultry-endemic lineage remained largely confined to Egypt, with only sporadic spillover to neighboring southwest Asia—likely mediated largely by poultry trade rather than migratory birds (Kim, 2018; Fasanmi et al., 2017).

### 3.3 2021–2022 H5N1 clade 2.3.4.4b epizootic in Eurasia and Africa: northward return

During the 2021–2022 clade 2.3.4.4b epizootic, viruses that had spread across Europe and reached Africa during the winter were detected again in Europe in the following spring and summer, including in seabird colonies (Briand et al., 2025). One illustrative example was the mortality event in Dalmatian pelicans (*Pelecanus crispus*) in Greece in early 2022, shortly after their return from wintering areas (Alexandrou et al., 2022, 2023). The timing and ecological context suggest infection acquired during migration or at southern stopover sites, followed by introduction into breeding colonies.

These observations do not by themselves demonstrate direct long-distance return of intact viral lineages, but they are consistent with the ecological plausibility of northward transport within Afro-Palaearctic flyways.

To place these events in context, it is necessary to consider the scale and diversity of avian influenza virus circulation within Africa. Surveillance data compiled by the FAO indicate that between 2017 and 2025 multiple H5 and H7 subtype viruses were detected across a wide range of African countries in both wild birds and poultry. H5N1 in particular showed broad geographic distribution across West, East, and Southern Africa, while other H5Nx subtypes, including H5N6 and H5N8, were reported in multiple regions. This widespread circulation provides conditions under which viruses may persist, diversify, and potentially contribute genetic material to viruses detected outside the continent.

The FAO reported that:

- Viruses simply as H5 (neuraminidase not specified) were confirmed in birds in Senegal, South Africa, Uganda and Libya.
- The subtype H5N1 was the most widely distributed and was reported from Côte d’Ivoire, Ghana, Nigeria, Burkina Faso, Niger, Cameroon, Ethiopia, Kenya, Egypt, Tanzania, South Africa, Senegal, Mauritania, Benin, Togo, Liberia, The Gambia, Mali, Sudan, Djibouti, Somalia, Angola, São Tomé and Príncipe, Mozambique, Malawi and Namibia.
- The subtype H5N2 was confirmed exclusively in South Africa.
- The subtype H5N6 was detected in Nigeria, Uganda, Benin, São Tomé and Príncipe, Côte d’Ivoire, Ghana, Togo, Burkina Faso, Niger, Senegal and Guinea.
- The subtype H5N8 was reported in South Africa, Cameroon, Nigeria, Burkina Faso, Niger, Ghana, Togo, Zimbabwe, Namibia, Côte d’Ivoire, Uganda, the Democratic Republic of the Congo, Lesotho and Zambia.

In addition to H5Nx viruses, highly pathogenic H7 viruses, including subtype H7N6, were confirmed in South Africa.

Importantly, FAO data indicate that since 1 October 2024, confirmed circulation of H5N1 clade 2.3.4.4b in sub-Saharan Africa has been restricted to Liberia, Niger, Nigeria and Togo, suggesting a substantial spatial contraction of active circulation of this lineage compared with earlier phases of the panzootic.

**Figure 7:**
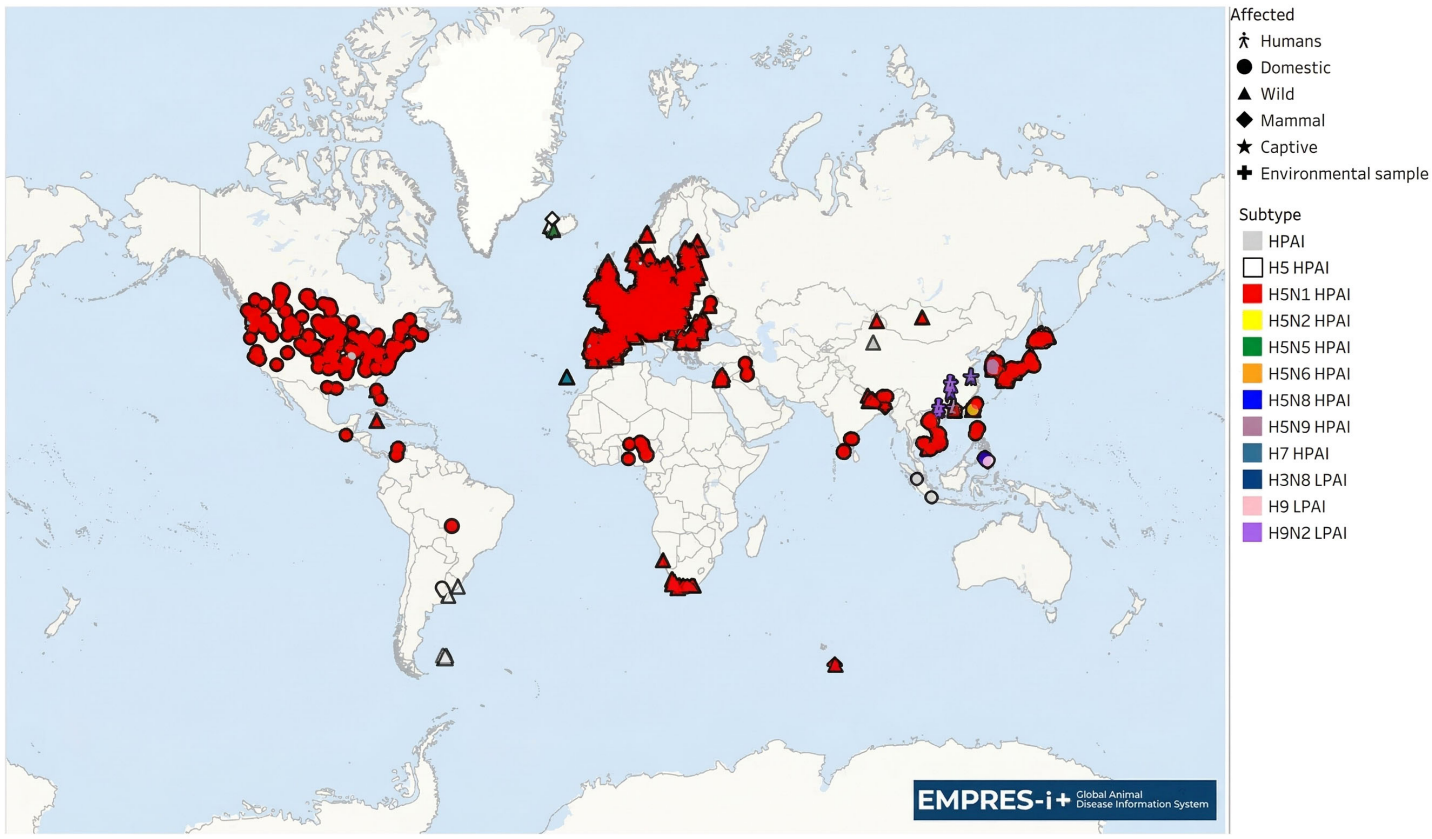
Locations of HxNy outbreaks observed globally as of February 2026. Food and Agriculture Organization (FAO), 2026.

FAO’s global charting of H5 outbreaks as of February 2026 thus indicates continuing presence in parts of West Africa (as indicated above) and southwest Asia, and a significant presence in Europe, although none in northern Eurasia and the Caspian Basin (Food and Agriculture Organisation (FAO), 2025) (**Figure 7**).

The FAO continues to state that the H5N1 clade 2.3.4.4b panzootic continues unabated its unprecedented global expansion, now affecting wild birds across six continents, causing outbreaks in poultry across dozens of countries, and spilling over into an expanding range of mammalian hosts including dairy cattle—the need for improved integrated surveillance and visualization tools has never been more crucial (Peacock et al., 2025).

The most recent WOAH global situation report, dated February 2025, states that the number of recorded outbreaks in wild birds for the first five months of that seasonal wave was comparable to that for the whole year of the previous wave, and that in Europe the number of outbreaks among wild birds has been increasing since January 2025. The situation is critically high (World Organisation for Animal Health (WOAH), 2025).

The European Food Safety Authority, European Centre for Disease Prevention and Control, European Union Reference Laboratory for Avian Influenza, December 2025, Avian influenza overview of December 2025, reports that between 6 September and 28 November 2025, 2,896 HPAI A (H5) virus detections were reported in domestic (442) and wild (2,454) birds in 29 countries in Europe. The magnitude and geographical extent of these detections were unprecedented for this time of the year, particularly in wild birds and secondary infections of poultry. Large numbers of waterfowl were affected by the disease, and mass mortality of common cranes (*Grus grus*) was observed along their migratory routes in Europe.

### 3.4 H5N1 clade 2.3.4.4b outbreaks in West African colonial waterbird systems (2020–2025)

**Figure 8:**
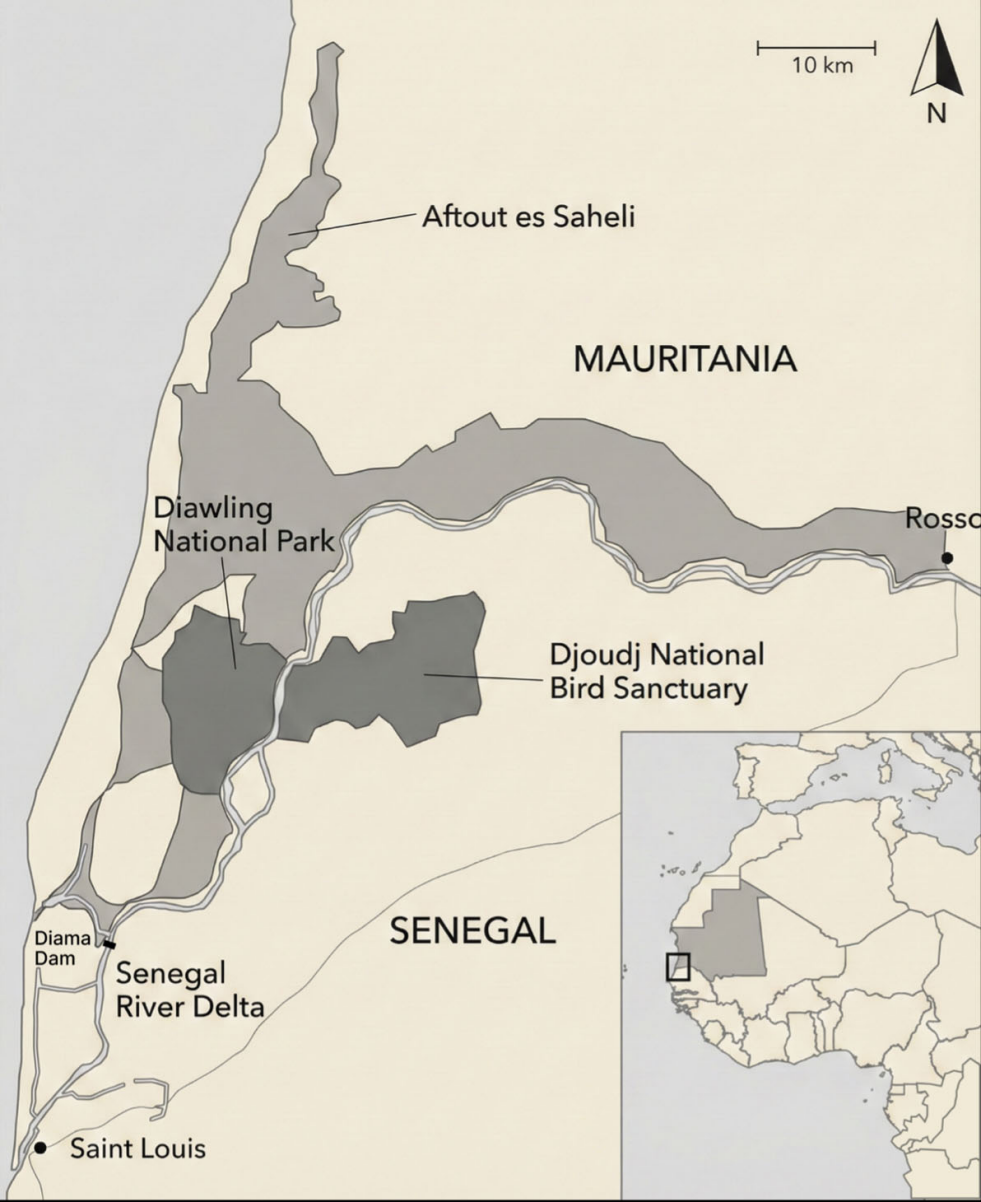
Sites of Djawling National Park (Mauritania) and the adjoining Djoudj National Bird Sanctuary (Senegal) on the Senegal River. Moreno-Opo et al., 2013.

Colonial waterbirds, including pelicans and terns, provide examples of how dense breeding aggregations can act as both recipient and amplification settings for HPAI. During December 2020–February 2021, a die-off of adult and juvenile great white pelicans occurred in Parc National de Diawling in the Senegal River Delta wetlands of south-west Mauritania (Beyit et al., 2023), totaling 2,140 birds (59 adults and 2,081 juveniles). In January 2021, Senegal also reported an outbreak in the Djoudj sanctuary with ~750 juvenile pelicans found dead from a population of ~8,800. The Mauritanian and Senegalese sites form part of the same greater wetland complex (including Aftout es Saheli) (**Figure 8**), which during the July–September rainy season supports ~220 bird species.

Prior to these events, there were no documented cases of H5N1 clade 2.3.4.4b in wild bird populations in these far West African countries, although other ancestral H5 lineages had been reported from poultry farms (Ducatez et al., 2007; Jatta et al., 2025). In early February 2022, a second outbreak with high mortality among great white pelicans occurred in Parc National de Diawling, affecting 58 adult birds, with concurrent impacts reported in Senegal’s Djoudj sanctuary; viruses were closely related to clade 2.3.4.4b strains circulating in Europe in 2022 (Beyit et al., 2023). These 2020–2022 pelican events represent among the first major wild-bird impacts of clade 2.3.4.4b in Africa, even as many West African countries had experienced other HPAI H5Nx incursions over the preceding two decades.

Most Djoudj pelicans are sedentary and remain in West Africa year-round, whereas another Eurasian population winters along Iran’s south Caspian coasts and breeds in Kazakhstan’s Turgai wetlands between western Siberia and the North Caspian, and at Lake Balkash in south-eastern Kazakhstan (Crivelli et al., 2000). The Mauritanian and Senegalese pelicans likely acquired infection from other taxa (e.g., dabbling ducks or other migratory waterfowl) that introduced H5N1, after which colony conditions (communal nesting and occasional kleptoparasitism) could facilitate rapid within-colony spread. The high mortality observed suggests great white pelicans are highly susceptible and may function largely as dead-end hosts for long-range viral transport in these settings.

In the North Caspian Sea, mixed colonies provide comparable multi-species transmission opportunities. During a May 2022 outbreak on Maliy Zhemchuzhniy Island, at least 14 Dalmatian pelicans reportedly died (Astrakhan Nature Reserve internal report, July 2025), although attribution to H5N1 was not formally confirmed. If due to H5N1, spillover from infected gulls and terns sharing the island is plausible. The same outbreak coincided with severe impacts on Caspian terns: >5,600 individuals died or failed to breed (adult mortality and egg abandonment). Caspian terns are long-lived, piscivorous, and some Caspian-breeding populations winter along the Indian Ocean and Persian Gulf; they nest densely and often alongside gulls on islands, facilitating transmission via scavenging and aggressive interactions.

Together, these events underscore a general mechanism: multi-species congregations—whether mixed seabird colonies or African wetland assemblages—create repeated opportunities for interspecific spillover, while species-specific movement patterns and susceptibility determine whether viruses are exported or amplified locally. The pelican and tern events were followed by a winter 2023/2024 mass mortality of swans at Lake Karakol near the eastern Kazakhstan Caspian coast, attributed to H5N1 clade 2.3.4.4b (Sultankulova et al., 2024).

### 3.5 West African seabird crisis: 2022–2023 HPAI H5N1 outbreak

Jatta et al. (2025) documented the largest recorded wild-bird mortality event in West Africa, based on a multi-site network of surveys across Senegal and Gambia. The outbreak showed a sporadic initial phase in late 2022 and an intense, laboratory-confirmed phase in 2023. See **Supplementary Material** (**SM Table 1**) for timeline and observed mortality during the West African seabird outbreak, and for species-specific mortality highlights reported for the West African seabird outbreak.

The outbreak’s timing coincided with the March–May breeding season, amplifying impacts on reproductively active adults at major colonies. Peak mortality occurred during dense pre-breeding aggregations and the cool dry season, conditions expected to prolong viral viability. The concurrent arrival of Palaearctic migrants during the boreal autumn likely facilitated introduction; phylogenetic analyzes linked outbreak strains to European H5N1 variants (Jatta et al., 2025). The cumulative impact was ~25,000 confirmed deaths across 32 species, compounding existing pressures from climate change and habitat degradation.

In April 2023, additional mass mortality among water and shore birds was recorded at Tanji Bird Reserve (Gambia) and in Senegal, attributed to HPAI H5N1 by the Dakar laboratory. WOAH also reported H5N1 in 752 wild birds (including terns, pelicans, cormorants and vultures) in the Kapatchez Delta and coastal wetlands of Guinea-Bissau. By mid-2023, A(H5) virus was detected in Nigeria among several wild species, including greater crested tern, pied crow and grey crowned crane (EFSA, 2025).

**Figure 9:**
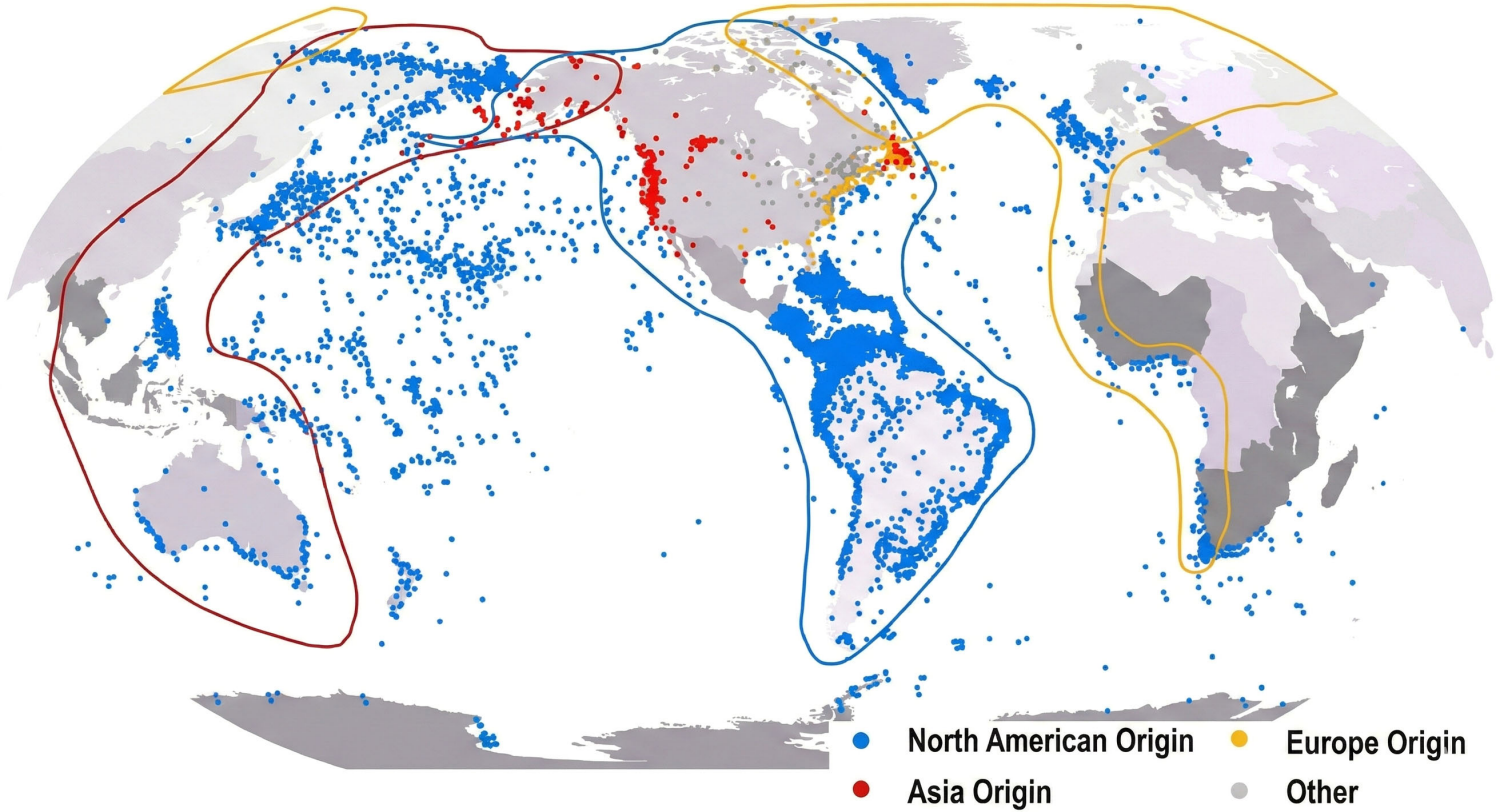
Distribution of bird bands for birds banded in North America and recovered in other continents. This map reveals the geographical extent of migratory bird movement between the western and southern African coasts and North America. The locations are restricted to ringed birds encountered less than 8 km from a continent’s coast. USDA, 2013.

An overlooked indicator of intercontinental connectivity is provided by a 2013 USDA assessment of wild-bird pathways for introduction of novel AIV into North America (focused on H7N9). The USDA infographic mapped bird band recoveries and highlighted migratory connectivity between the western and southern African coasts and North America; the analysis was restricted to ringed birds encountered less than 8 km from a continent’s coast (**Figure 9**).

### 3.6 Entire Palaearctic Region: H5N8 multi-focal outbreak and new H5N1 variants (2020-2021)

A global outbreak of a new H5 variant affected poultry and wild birds in Russia and across the wider Palaearctic region in 2020-2021 (Lublin et al., 2023). From October 2020, reassortment between clade 2.3.4.4b H5N8 (contributing the HA glycoprotein) and low-pathogenic avian influenza (LPAI) viruses from wild birds in Asia and Europe (contributing the NA glycoprotein) generated multiple H5Nx reassortants in Europe, including H5N1, H5N2, H5N3, H5N4, H5N5 and H5N8 (Lublin et al., 2023).

These European and Eurasian clade 2.3.4.4b H5N1 viruses followed the 2020 H5N8 wave that seeded the H5 hemagglutinin into multiple reassortant constellations (including N1). By the end of 2021, the new AIV-H5N1 viruses had become predominant in Asia, Africa and Europe, and the 2021–2022 HPAI season was the largest so far observed in Europe (Xie et al., 2025).

## 4 Caspian Basin/northern Eurasia-Africa avian influenza links

### 4.1 Caspian Basin-Africa avian influenza viral links

From the perspective of avian influenza virus evolution and inter-regional transfer, researchers at the Federal Centre of Animal Health (Vladimir, Russia) reported H5N5 viruses isolated from birds sampled in the northern Caspian region in 2020-2021 (Zinyakov et al., 2022). These isolates, collectively referred to as Caspian Region Viruses (VCR), are:

A/dalmatian pelican/Astrakhan/417-2/2021(H5N5) & A/dalmatian pelican/Astrakhan/417-1/2021(H5N5) (both recovered 1 April 2021); A/pelican/Dagestan/397-1/2021(H5N5) & A/gull/Dagestan/397-2/2021(H5N5) (both recovered 6 April 2021; A/waterfowl/Russia/1526-4/2021(H5N5) [recovered 23 September 2021]; and A/shelduck/Kalmykia/1814-1/2021(H5N5) [recovered 8 November 2021].

The Caspian Basin and Western Europe are geographically linked parts of the Eurasian Palaearctic and are ornithologically connected by substantial bird movement. Birds moving westward or northwestward from the Caspian/Black Sea region enter Europe’s eastern periphery and may continue into Western Europe; others move south via the Black Sea-Mediterranean flyway (Najdenski et al., 2018). Because many waterbirds stage or winter around the Caspian but occupy Europe during other parts of their annual cycle, influenza viruses can flow along this continuum.

Multiple European epizootics illustrate this connectivity. Following infection of wild birds at Qinghai Lake in 2005, H5N1 spread west through Kazakhstan and Russia (north of the Caspian). By summer-autumn 2005, H5N1 was detected in Western Siberia (De Marco et al., 2014), the Volga/Caspian and the Urals, and by October 2005 it had reached Turkey, Romania and other parts of Eastern Europe. Thus, the Caspian served as a springboard for H5N1 into Europe. Although clade 2.2 was sometimes termed the “Qinghai strain”, it could equally be viewed as a ‘Caspian/Black Sea strain’ by the time it affected Europe, because that was the immediate source region.

The Caspian/Black Sea sector has likely played a similar relay role for later H5Nx viruses. The 2016 H5N8 clade 2.3.4.4b epizootic likely involved wild birds carrying the virus from breeding grounds in Siberia to the Caspian/Black Sea region and then into Europe (Sobolev et al., 2021). By early October 2016, H5N8 was noted in birds around the Ural region (on Russia’s eastern European perimeter), quickly followed by outbreaks in Hungary, Germany and elsewhere in Europe; many early-2016 European H5N8 isolates were closely related to isolates from wild birds in Russia.

In 2021, H5N1 clade 2.3.4.4b surprisingly persisted through summer in Europe, possibly maintained in resident birds or continually reintroduced by movements within Eurasia (Fusaro et al., 2024). That autumn (2021), Europe experienced an even larger H5N1 outbreak, and it has been suggested that birds migrating from northern Russia (where they had mixed with birds from Asia) introduced fresh viral diversity. Because the Caspian Basin lies on the route of many Siberian breeders migrating to Europe, it is plausible that some of those birds acquired or shed virus there before continuing westward.

Even outside major HPAI episodes, routine low-pathogenic avian influenza (LPAI) exchange between the Caspian region and Europe occurs. A review of global wild-bird influenza patterns by Olsen et al. (2006) noted that inter-regional exchange in Eurasia occurs particularly where migratory zones overlap; the Caspian and Black Sea zone is one such overlap where Asian and European bird populations intermingle, allowing Asian-origin viruses to enter European bird communities.

Movement is bidirectional. Many waterbirds breeding or summering in Europe migrate south-east to the Caspian Sea for winter (Petherbridge et al., 2022), so viruses circulating in those birds in Europe can be introduced into the Caspian Basin during autumn migration. Birds that breed in Europe but winter in, for example, India or East Africa may also pass through Caspian stopovers, carrying infections acquired in Europe. After Europe’s peak in early 2006, H5N1 was detected in spring in Azerbaijan (with four human fatalities; Gilsdorf et al., 2006; Azerbaijan: avian influenza, 2006) and later in 2006 in southern Russia, consistent with viruses introduced from Europe. Once in the Caspian/Southwest Asia area in spring, some H5N1 persisted in resident birds or poultry throughout summer (one introduction into Egypt in 2006-07, for example, occurred slightly later and may have involved that route) (Aly et al., 2008; Abdelwhab et al., 2010; El-Shesheny et al., 2023).

The H5N1 clade 2.3.4.4b that devastated Caspian breeding colonies in 2022 was already rampant in Europe in late 2021. It is plausible that infected birds from Europe (e.g., juvenile gulls or ducks) moved to the Caspian over winter or early spring, bringing the virus with them. Where wild and domestic bird populations intermingle, HPAI viruses may settle into an endemic cycle. For example, H5N1 (and H9N2) became entrenched in poultry in parts of Southwest Asia (Egypt, Iran) (El Shesheny et al., 2012; Kayali et al., 2011, 2016; Monne et al., 2013; Gomaa et al., 2015; El-Shesheny et al., 2023; Emami and Ghalyanchi Langeroudi, 2022) and South Asia after introduction via migratory birds, and the Caspian countries sit at a migratory nexus of these regions. A virus can overwinter in domestic ducks around the Caspian, then infect wild birds in spring which carry it away; conversely, viruses picked up in breeding areas may be carried to Africa in autumn by naive juveniles and adults migrating south. This two-way exchange is fundamental to understanding how HPAI H5N1 circulates between Caspian and African bird populations.

Additionally, along the Caspian coast some waterbirds now overwinter there instead of migrating further south, likely in response to milder winters. Such conditions may allow certain HPAI strains to persist locally (‘oversummer’) and support low-level virus circulation outside the main migration seasons.

As a key hub in Eurasian bird migration systems that enables intercontinental exchange, genetic reassortment and further spread of avian-borne viruses, the Caspian region remains a locus of particular panzootic concern for H5N1 clade 2.3.4.4b.

### 4.2 Under-researched migratory flyway/AIV transmission route from Europe and the Caspian Basin to the African Sahel via the Sudd Wetlands in South Sudan

**Figure 10:**
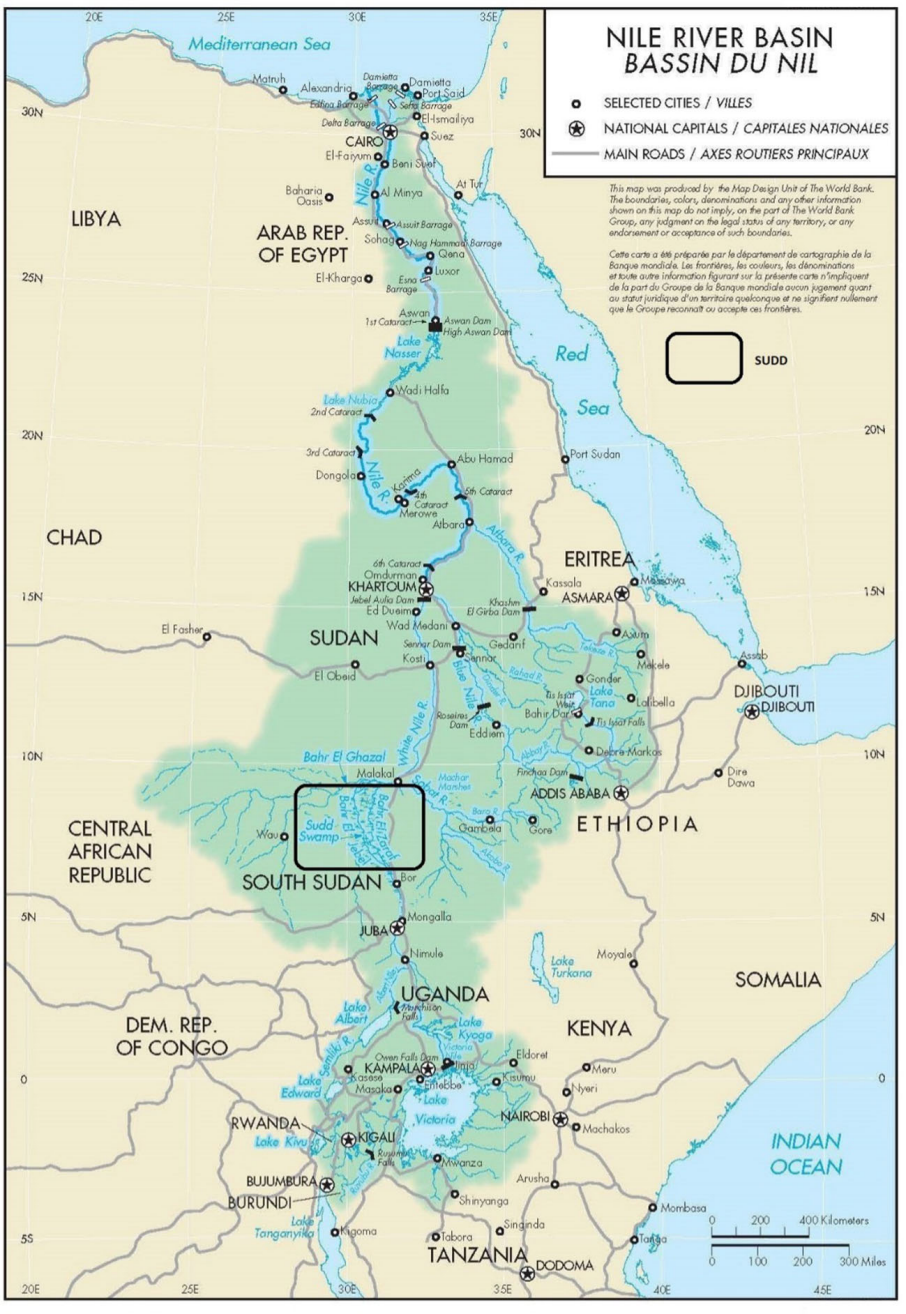
Geographic location of the Sudd wetlands on the White Nile River, South Sudan. World Bank.

Large wetlands along the Caspian (deltas, coastal shallows, lagoons) host huge concentrations of migratory waterbirds as breeding sites, stopovers and wintering areas. Many birds that breed in Western Siberia and Central Asia migrate along the western littoral of the Caspian Sea into Southwest Asia (a major migratory corridor lies between Israel and Jordan) (Frumkin et al., 1995) and then either migrate toward Lake Chad (now in regression) and the West African wetlands, or follow the Nile upstream via the Sudd wetlands of South Sudan (**Figure 10**).

**Figure 11:**
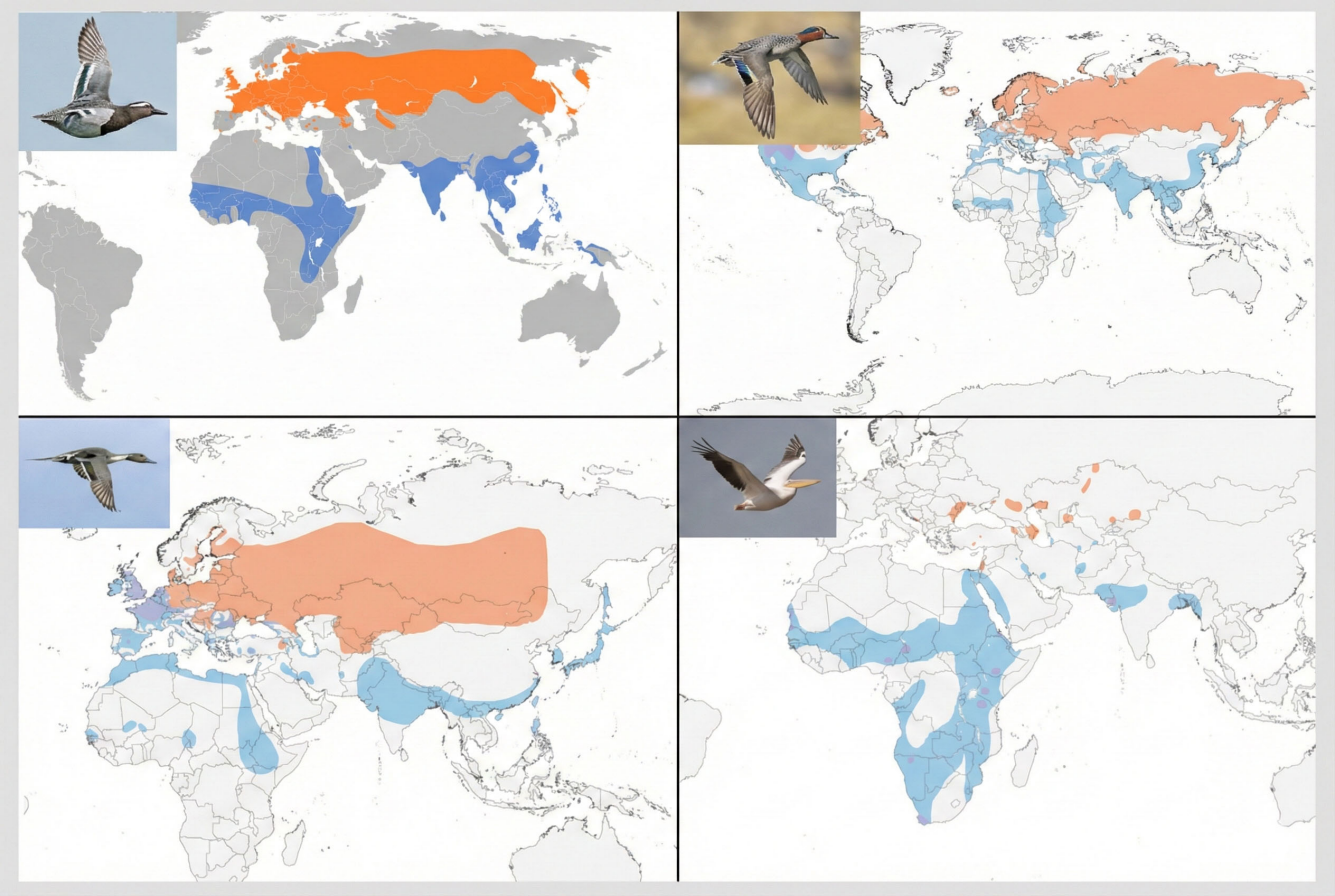
**(A–D)** World distribution maps of the garganey (*Spatula querquedula*), Eurasian teal (*Anas crecca*), northern pintail (*Anas acuta*) and Dalmatian pelican (*Pelecanus crispus*) showing migration routes along the Nile to the Sudd wetlands and thence to Central and West Africa. Lynx Edicions/BirdLife International, 2014.

The Sudd is among the most extensive wetlands in the world after the wetlands of western Siberia (MoEF, 2022). However, it has been largely overlooked in avian influenza discussions as a migratory route because of ongoing conflict, emigration of expert local virologists, and a public-health focus on avian influenza outbreaks in poultry. Ornithological evidence for Nile-Sudd-Sahel connectivity is nevertheless strong: the nonbreeding range map of garganey (the most abundant Eurasian waterbird migrant to West Africa) supports this corridor (**Figure 11a**), and analogous nonbreeding ranges are shown for Eurasian teal (**Figure 11b**), common pochard, northern pintail (**Figure 11c**) and northern shoveler. Dalmatian pelican also follows a broadly similar route, across a wider front and farther south (**Figure 11d**) (BirdLife Data Zone).

Despite limited phylogeographic data for these species in this region, these distributions imply that the Sudd Basin may function as a dynamic African nexus for AIV, analogous to the Caspian Basin in Eurasia. Supporting this possibility appears to be the Twabela et al. (2018) report of detection in 2017 of H5N8 clade 2.3.4.4b in both farmed poultry and wild ducks (species not identified) in the Democratic Republic of the Congo, and provision of a whole-genome phylogenetic tree indicating possible genome transfer between the Caspian Basin and northern Eurasia.

In late 2016, a reassortant HPAI H5N8 virus (clade 2.3.4.4 group B) began to spread from China and the Russian Federation to Asia, the Middle East, Europe and western Africa and for the first time reached central, eastern and southern Africa. Egypt, Tunisia and Nigeria reported HPAI H5N8 virus in late autumn 2016, and virus detection continued to occur across Africa in the winter, spring and summer of 2017.

Phylogenetic relationships of influenza viruses detected in the Caspian region (Astrakhan Region and the Republic of Dagestan) with viruses from Israel and Romania indicate spread of the H5N1 clade 2.3.4.4b virus along the West Asian-East African and Black Sea-Mediterranean flyways (Sobolev et al., 2023). Furthermore, the presence of segments in the genomes of Caspian low- and highly pathogenic influenza viruses related to Asian strains (from Siberia, the Far East, Mongolia and China) also demonstrates the role of the Central Asian flyway in the spread of influenza viruses through and beyond the Caspian region (Sobolev et al., 2023; Murashkina et al., 2024).

## 5 Dynamics of avian host species viral linkages and virus transmission structures

### 5.1 Host species linkages and virus transmission

Ecological relationships among waterfowl species strongly influence avian influenza virus (AIV) transmission pathways. In both the Caspian and African wetlands, gulls, ducks and pelicans often share habitat and interact closely (during breeding, molting, staging and wintering), creating frequent interfaces for interspecies viral spread.

AIV circulate naturally in wild waterbirds of the orders Anseriformes (ducks, geese, swans) and Charadriiformes (gulls, terns, shorebirds). Caspian species of these groups therefore act as reservoirs for diverse AIV subtypes (particularly low-pathogenic strains such as H13 and H16 in gulls, and H4-H6, H11 etc. in ducks). Periodically, HPAI strains emerge from the A/Goose/Guangdong/1/96 (Gs/GD) lineage H5N1 (first detected in 1996) and spread along migratory routes. The H5N1 HPAI lineage has diversified into multiple clades, of which clade 2.3.4.4b has caused panzootic outbreaks since 2020.

During the 2020–2021 H5N1 wave in Europe, numerous infections were detected in apparently healthy wild ducks, indicating that they can harbor and disperse HPAI without conspicuous mortality. Ducks likely introduced the virus into West Africa: phylogenetic analysis of H5N1 strains from Senegal clustered them with European wild-bird lineage viruses from late 2020, suggesting migratory waterfowl carried the virus to Senegal’s wetlands. The estimated most recent common ancestor (approximately November 2020) coincided with southward migration (Lo et al., 2022).

Among probable vectors, garganey and northern pintail migrate from Eurasia to West Africa and were present in huge numbers in Senegal and Mali at that time (Roux and Jarry, 1984; Koehler et al., 2008). These ducks mingle with resident species and other migrants at sites such as Djoudj, potentially seeding infections in more sedentary birds. Ducks are often tolerant to H5N1 but can shed large amounts of virus, contaminating shared water and soil. In the Nile Delta (Egypt), migratory waterfowl similarly intermingle with resident birds and domestic poultry, facilitating cross-species transmission (Naguib et al., 2019). Thus, ducks act as intermediate hosts connecting distant regions by transporting viruses during migration and seeding outbreaks among other wild birds (and occasionally poultry) at stopovers and wintering sites.

### 5.2 Dynamics of viral transmission, reassortment and reticulate evolution across Western Palaearctic and African sectors of global HxNy viral populations

Viruses have circulated among wild aquatic birds for millennia, are continually evolving (USDA, 2013; Shao et al., 2017) and are extremely mobile, if there are receptive vectors (Chen and Holmes, 2006, 2009). An avian influenza virus is therefore not simply moved across the Caspian Sea by birds; it can also change rapidly as genetic variants from different directions enter the region and re-associate (Dugan et al., 2008). Viruses have fast replication cycles, so variation and selection can operate quickly. They require much less time to replicate than either bacteria or animal cells, creating more opportunities for the viral genome to change and more opportunities for selection. This infection dynamic drives constant evolutionary change at the virus-host interface (Alkie et al., 2023).

Given the uncertainties and obstacles to AIV transmission among multiple hosts and across biogeographic barriers, it remains difficult to predict where, when and which influenza strains will emerge and which hosts will be affected (Fusaro et al., 2024). Any assessment of the likelihood that a specific virus would be carried within and between continents via aquatic birds (or another pathway) is limited by available data. Available data show that aquatic birds can move AIV strains largely unaltered over long distances, but this appears to be rare. More commonly, segments of the AIV genome appear in new regions (including intercontinentally) through reassortment events occurring along migratory flyways (USDA, 2013; Campitelli et al., 2008; Hill et al., 2022).

A simple linear model of virus spread along a single path is therefore often insufficient. Previously obtained genetic data on Caspian viruses indicate mosaic genome structures (Murashkina et al., 2024). In such cases, the phylogeography of reassortant viruses may be better represented as reticulate rather than strictly hierarchical, with the Caspian region acting as one of the main nodes of this network and a source of new hybrid genotypes (through reassortment of segments) with traits that may facilitate further distribution (Makarenkov and Legendre, 2004; Chan et al., 2013; Gontier, 2015; Bjornson et al., 2024). Geographic space and evolutionary time interact: lineages may break apart and then re-form into slightly different units, creating a reticulate pattern in both space and time (Chan et al., 2013; Veron, 2011; McBreen and Lockhart, 2006; Nakhleh et al., 2005; Zou et al., 2024).

Reticulation network approaches have also been applied to the evolution of avian influenza in swine. Establishment of non-swine AIV viruses and gene segments in swine has altered epidemiology to such an extent that all swine AIVs circulating in the USA contain genes derived from reassortment between swine-, human- and avian-origin viruses (Macken et al., 2006; Ma et al., 2016; Ma, 2016; Markin et al., 2019; Makarenkov et al., 2004; Morgan, 2016; Bjornson et al., 2024; Nelson et al., 2015).

### 5.3 Southward (and return) flow Caspian Basin - Africa: contribution to H5Nx’s spread into Africa (2005-2006)

Evidence of AIV flow from the Caspian region to Africa overlaps with the Europe/Eurasia-to-Africa patterns described earlier, but with a more specific geographic locus. In phylogeographic reconstructions, many virus incursions into Africa in 2005–2006 are mapped back to the vicinity of the Black Sea, which is connected to the Caspian via migratory routes and includes regions just west of the Caspian (e.g., southern Russia and the Caucasus).

Accordingly, migratory waterbirds moving south from the northern Caspian/Volga catchment region in autumn 2005 likely carried H5N1 toward Africa. The first HPAI outbreak recorded in the Caspian Sea region was in late 2005, when at least 400 wild mute swans died of H5N1 on the Volga Delta (North Caspian) (Lvov et al., 2006). This occurred just months before the virus appeared in Nigeria, suggesting an unbroken chain of transmission southward along overlapping flyway zones. Waterbirds infected in the Volga-Caspian area in autumn could traverse Southwest Asia or northeastern Africa within weeks.

Many Anseriformes from the Caspian migrate to the Nile Delta and East Africa. For example, northern pintails tagged in West Siberia have been known to winter in Sudan and East Africa, and garganey (which congregate on the Caspian during migration) winter in huge numbers in both West and East Africa. The 2005–2006 H5N1 strain likely followed such pathways. Similarly, for the 2016–2017 H5N8 wave, phylogeography has pointed to north-central Asia (including regions around the Caspian Sea) as a direct source for some African introductions: H5N8 detected in West Africa did not always match Western European viruses, but rather viruses from Russia, indicating that some birds may have flown south from breeding areas in Russia (Xiong et al., 2021).

Certain populations of garganey and ruff (a wading bird*, Calidris pugnax*) migrate from West Siberia across the eastern Mediterranean to West Africa, largely skipping Europe. If these populations carried H5N8, they would produce the observed pattern of West African H5N8 strains being closely related to Siberian ones. The West Asia-East African flyway zone and the Black Sea-Mediterranean flyway zone both encompass the Caspian/Transcaucasus region and then diverge in the northern Egyptian wetlands toward West or East Africa (via the White Nile). In 2016, both routes likely delivered H5N8: one via Europe/Caspian and another via West Africa from Caspian/Central Asia. The Caspian also indirectly contributed to clade 2.3.2.1c H5N1 in 2014, with limited sequence data suggesting a source in Southwest Asia and the area surrounding the Caspian and Black Seas for the strain that reached West Africa (Fusaro et al., 2019).

A return flow of AIV from Africa back to the Caspian Basin is plausible and likely occurs for the same reason as documented by Pohlmann et al. (2022) for Africa-to-Europe movement: many birds that winter in Africa return each spring to breeding areas around and north of the Caspian. If they contract infection in Africa late in the wintering season, they can introduce it into Caspian nesting colonies. Large mixed-species colonies (gulls, terns, pelicans and ducks nesting in close proximity) provide abundant opportunities for interspecies transmission and viral reassortment.

**Figure 12:**
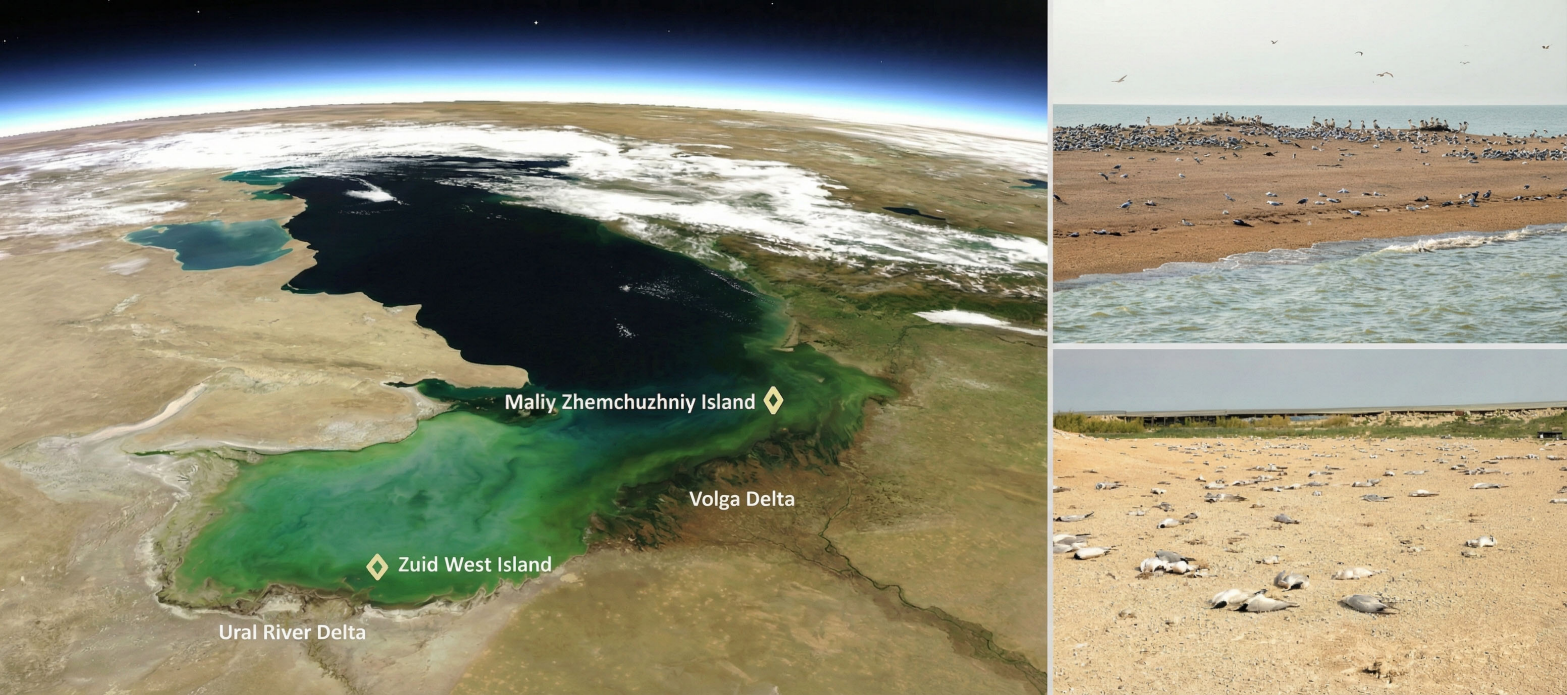
**(A)** Satellite image of north Caspian Basin. Indicated are the Volga Delta and the locations of the seabird breeding sites of Maliy Zhemchuzhniy Island (Russia) and Zuid West Island (and nearby islets) (Kazakhstan) affected by the 2022 panzootic. NASA Terra Satellite 11.08.2014. **(B)** Maliy Zhemchuzhniy Island (northwest Caspian Sea, Russia) breeding colony of Caspian gulls (*Larus cachinnans*) and Dalmatian pelicans (*Pelecanus crispus*) during the 2022 H5N1 clade 2.3.4.4b panzootic. Note dead and dying birds in foreground. Astrakhan State Nature Biosphere Reserve, 2022. **(C)** Caspian tern (*Hydroprogne caspia*) and great black-headed gull *(Ichthyaetus ichthyaetus*) mortalities from the same 2022 panzootic as in **Figure 12B)**. Zuid West Island (Akzhayk State Nature Reserve), Kazakhstan waters of North Caspian Sea. Kydyrmanov et al., 2024.

A dramatic illustration was the mass mortality in May-June 2022 on islands in the north Caspian Sea (**Figure 12a**). One outbreak occurred on Maliy Zhemchuzhniy Island (North Caspian Sea), where over 30,000 waterbirds (Caspian terns, *Hydroprogne caspia*; Caspian gulls, *Larus cachinnans*; and great black-headed gulls, *Ichthyaetus ichthyaetus*), died of HPAI H5N1 (**Figure 12b**). The virus was confirmed as clade 2.3.4.4b H5N1, the same highly virulent strain circulating in Europe at that time (Sobolev et al., 2023), and the same clade was confirmed for mass mortality on Kazakhstan Caspian Sea islets at the same time (Kydyrmanov et al., 2024) (**Figure 12c**). One hypothesis is that migratory waterbirds carried H5N1 from wintering grounds (perhaps in Africa or the Indian subcontinent) back into the Caspian ecosystem in spring 2022, seeding outbreaks when colonial breeders aggregated.

Caspian terns are migratory; many from Eurasian breeding colonies winter in East Africa or South Asia. If even a few terns or gulls returned infected (or contacted infected birds en route), they could spark an epidemic in the dense breeding aggregations on Caspian islets. While direct proof of an African origin for the 2022 virus is circumstantial, the same H5N1 strain was widespread in West and East Africa during winter 2021-22, and northward movement in spring could have occurred via birds coming from African sites through the Israel/Jordan corridor (Lublin et al., 2023). More generally, any LPAI viruses acquired in African wetlands may be carried back to the Caspian by migrants that breed in Central Asia; although the Caspian hosts many resident or short-distance migrants, it also hosts transcontinental migrants such as garganey.

A 2018 study on AIV in wild birds from the Caspian coast (Dagestan) found a range of LPAI subtypes (H1N1, H3N8, H7N3, etc.), all belonging to typical Eurasian lineages with no unique local pattern (Murashkina et al., 2024). This implies continual refreshment from migrating birds rather than isolated ‘Caspian-only’ lineages. Some of these viruses could have originated in Africa or in birds that had been to Africa, given that Palaearctic-African migrants contribute to the Caspian avifauna.

### 5.4 Virological evidence of ancestry and reassortment linking Africa, Southwest Asia and the Caspian Basin

That the Northeast African-to-Caspian sector of the merged flyway zone can function as a transmission route for avian influenza was evidenced as early as 2007 by Salzberg et al.

Human cases of H5N1 infection were reported beginning in January 2006 in Egypt, Iraq, Turkey, Djibouti and Azerbaijan. To better understand the ecology and epidemiology of this highly pathogenic virus in its transcontinental spread (predominantly from a public health perspective), Salzberg et al. (2007) sequenced and analyzed complete genomes of 36 then-recent influenza A(H5N1) viruses collected from birds in Europe, northern Africa and southeastern Asia (see **Table 3**).

**Table 3:** Salzberg et al. (2007): key phylogenetic findings relevant to Europe-Africa-Caspian connectivity.

Key findings of Salzberg et al., 2007	Details
Dataset and scope	Complete genomes of 36 influenza A(H5N1) viruses collected from birds in Europe, northern Africa and southeastern Asia; among the first complete H5N1 genomes generated outside Asia; used to depict lineages infecting wild and domestic birds and their relationships to other strains affecting birds and humans.
Major phylogenetic structure	Isolates fell into 3 distinct lineages, 1 of which contained all known non-Asian isolates (Euro-African lineage).
Introductions and diversification in Europe-Africa	Euro-African lineage caused several fatal human infections in Egypt and Iraq in 2006; introduced at least 3 times into the European-African region, splitting into 3 distinct, independently evolving sublineages; 1 isolate provided evidence that 2 sublineages had reassorted by 2007; European countries were affected by each of the 3 introductions.
EMA-2 sublineage and inferred source region	EMA-2 contained 1 European isolate (swan, Croatia) and multiple isolates from domesticated birds in Nigeria and Niger; shared a common ancestor with isolates from Astrakhan and Kurgan (Russia). H5N1 isolates from Europe, the Middle East and Africa were closely related despite wide dispersal, including Cote d’Ivoire, Nigeria, Niger, Sudan, Egypt, Afghanistan, Iran, Slovenia, Croatia and Italy. Shared lineage suggested a single genetic source for introduction into western Europe and northern and western Africa; the most recent source was placed in either Russia or Qinghai Province in China.

Molecular epidemiology continues to show specific genetic connections between avian influenza viruses detected in Caspian birds and those in Africa/Egypt. The ongoing H5N1 clade 2.3.4.4b panzootic is characterized by extensive reassortment (exchange of gene segments when a host is co-infected with different influenza strains). Mixed-species wild-bird gatherings provide favorable conditions for reassortment and the emergence of new genotypes (here, a genotype is a particular constellation of H5N1 gene segments). Surveillance in Europe has documented an explosion of H5Nx genotypic diversity since 2020: at least 50 new genotypes of HPAI H5 were observed in Europe from 2020–2022 alone (Dupas et al., 2026). Many arose from clade 2.3.4.4b viruses reassorting with locally circulating LPAI viruses (often adapted to wild duck or gull hosts), potentially yielding fitness advantages such as improved replication in particular hosts or persistence across seasons (Hoye et al., 2010).

### 5.5 Egyptian-lineage AIV virus in North Caspian Volga Delta gulls (2020)

Episodes in the Volga Delta and north Caspian illustrate how avian influenza lineages and individual gene segments can move bidirectionally between Africa/Southwest Asia and Eurasia, and reassort at flyway mixing nodes (see **Supplementary Material** (**SM Table 2**)).

In 2020, not long after clade 2.3.4.4b H5N1 first struck Europe, an H5N8 variant with genetic segments belonging to the Egyptian lineage was detected in black-headed gulls and waterfowl in Russia’s Volga Delta, a key waterfowl breeding area of the northwest Caspian (Sobolev et al., 2023). Genetic analysis suggested that this virus likely descended from H5Nx viruses circulating in the Nile Delta during 2017-2019. Egypt has been endemic for an older H5N1 clade 2.2.1 in poultry; by 2016–2017 it also experienced incursions of clade 2.3.4.4b H5N8 outbreaks in domestic birds and continued circulation in wild birds (El-Shesheny et al., 2021). We suggest that an H5N8 virus from Egypt or Southwest Asia spread northwards via migrating waterfowl in early 2020, reached the Volga-Caspian region, and reassorted with Eurasian LPAI viruses, generating at least two distinct genotypes. By autumn 2020, both the original Egyptian-like strain and a reassortant strain dispersed westward into Europe. Notably, one genotype was an H5N1 strain resulting from reassortment between the incoming H5N8 and local avian viruses, and this emergent H5N1 was detected in wild birds in the Netherlands and elsewhere in fall 2020.

Black-headed gulls and other waterfowl in the Volga Delta were among the first recognized wild hosts, with mass mortality events and virus isolations reported during 2020 (including contemporaneous reports documenting H5N8/H5N1 in waterfowl in the Astrakhan region; Pyankova et al., 2021). Field reports are sparse, but the large breeding colonies (tens of thousands of gulls) likely became infected via contact with infected waterfowl and/or predation on sick waterfowl and chicks. Russian virologists dubbed one novel genotype ‘EA-3’ (Eurasian-African reassortant 3), reflecting its mixed origin. Genomic sequencing of 2020 Caspian-region isolates showed signatures of Egyptian origin in the HA segment (grouping with Egypt’s 2017–2019 H5N8 strains) and in the matrix (M) gene, whereas other segments (PB2, PA, NP) clustered with contemporary Eurasian wild-bird viruses and even with European H5N1 sequences from late 2020. This mosaic genome provides evidence of inter-regional viral ancestry. In summary, the Volga Delta episode exemplifies how a virus originating from an outbreak in Egypt’s poultry/wild birds can be carried by migrating birds to the Caspian and reassort there, spill into gulls, and subsequently seed the winter 2020–21 outbreaks in Europe and beyond to the Americas.

By winter 2021-2022, clade 2.3.4.4b H5N1 was causing widespread outbreaks in Europe and had also been detected across multiple African countries. The January 2021 Senegal events in pelicans and poultry were followed by H5N1 reports in Mauritania, Mali, Niger and Nigeria in 2021. Although genetic data from some African outbreaks are limited, in Senegal and Mauritania the viruses were found to be closely related to late 2020 European strains. (Ndao, 2023). After March 2023, thousands of birds were reported dead along the coasts of Senegal, Gambia, and Guinea-Bissau. By late 2021, some African-propagated viruses could therefore have been carried north by spring migratory birds. (Birdlife International, 2024; CMS FAO Co-convened Scientific Task Force on Avian Influenza and Wild Birds, 2022). Whole-genome sequencing of H5N1 isolates from dead Caspian terns during the May 2022 mass mortality on Maliy Zhemchuzhniy Island showed that one of the virus’s eight gene segments had a clear African origin: the PA (polymerase acidic protein) gene was closely related to H5N1 strains identified in Africa.

The most likely source of this PA segment is the West African lineage from 2020-2021, given the timing. The north Caspian 2022 outbreak strain was therefore a reassortant: HA, NA, PB1, M (and perhaps other segments) were derived from European lineage viruses circulating in Europe and the Middle East in winter 2021-22, whereas the PA segment was imported from a virus that had circulated in Africa. Investigators hypothesized that the ancestor of the Caspian strain overwintered in 2021–22 somewhere in Southwest Asia (possibly Israel) and was introduced to breeding colonies during spring migration (Sobolev et al., 2023). It remains unclear whether the African-derived PA segment reached the Caspian via birds travelling Africa -> Southwest Asia -> Caspian, or whether reassortment occurred at an intermediate location (e.g., Israel or Turkey). Israel experienced large H5N1 outbreaks in wild birds and poultry in late 2021, including a massive die-off of common cranes (*Grus grus*) in December 2021 (Lublin et al., 2023; Grinchenko et al., 2018), providing a plausible mixing environment. Detection of African-origin genetic material in the 2022 north Caspian isolates therefore provides genomic proof of south-to-north viral gene flow and supports a bidirectional exchange model.

Virologically, incorporation of an African-lineage PA segment implies co-infection with African- and Eurasian-lineage viruses at some point along the flyway, allowing segments to mix. The host could have been a migratory species visiting both regions (e.g., a duck or wader wintering in West Africa and migrating to Eurasia in spring) or a sequence of hosts along the route with stepwise movement and reassortment en route. The outcome may have been the highly virulent H5N1 genotype that caused one of the worst wild-avifauna die-offs recorded in the Caspian. Overall, the evidence indicates two-way exchange: European-origin H5N1 viruses reached Africa via migratory waterfowl in 2020, and African-lineage segments from 2021–22 West African outbreaks were carried back into Eurasian routes, seeding reassortant H5N1 in Caspian colonies by 2022.

Collectively, these observations point to an ecologically coupled network of AIV transmission between the Caspian region and Africa, with gull-duck-pelican interactions at key wetlands enabling both long-distance spread and bidirectional gene flow during the evolving panzootic.

## 6 Discussion

The ancestry and movement of H5N1 and other H5 avian influenza viruses between the Caspian Sea Basin, Africa and Europe are best understood as a product of migratory bird ecology. Virological data show a pattern of reciprocal exchange. This study has traced bidirectional H5 viral flows from 2006-2023, which epidemiologically link three principal regional dualities of the Palaearctic-Afro-Tropical flyways: (1) Caspian Basin-Africa/Africa-Caspian Basin, (2) Europe-Africa/Africa-Europe and (3) Caspian Basin-Europe/Europe-Caspian Basin. In turn, across the Atlantic from Northwestern Europe HPAI H5N1, which has been generated within this virological feedback loop, has been transmitted to North America, there to reassort and develop multiple new genotypes within native wildlife, domestic and farmed animals. Thence the virus has spread to the Antarctic mainland.

The Caspian Sea’s waterbird colonies – home to species migrating from Europe, Asia, and Africa – have emerged as a crucible where AIV genes from various sources mix. In 2020, an HPAI H5 virus originating from Egyptian outbreaks was introduced to Caspian waterfowl, possibly presaging a major wave of H5N1 in Eurasia. By 2022, after cycling through wild populations in Africa, the virus came full circle: Caspian birds were struck by H5N1 carrying an African lineage segment. Such bidirectional flow was not widely documented in earlier H5N1 epizootics and signals a new chapter in our understanding of the virus’s evolution. It suggests that H5N1 is becoming embedded in the wild bird ecosystem on multiple continents, with gulls and other colonial seabirds playing as significant a role as ducks in maintenance and spread.

That the Northeast African to Caspian Basin sector of the merged flyway zone was a transmission route for new avian influenza genotypes was evidenced as early as 2007 by Salzberg et al.

Pohlmann et al. (2022) have demonstrated, new substantive incursions of a second sublineage of HPAI H5N1 viruses (B2) have been detected since October 2021 showing an African ancestral relationship in their HA genes but was also detected contemporaneously in a wider area, including poultry outbreaks in Russia in September 2021. H5 sequences from Senegal and Nigeria (February to March 2021) are in the younger ancestry of this lineage, although its eldest ancestors were detected in Italy in November 2020. Świętoń et al. (2020) reported detection of a highly pathogenic avian influenza A(H5N8) clade 2.3.4.4b virus in Europe, which was generated by reassortment between a H5N8 subtype virus from sub-Saharan Africa and low pathogenicity avian influenza viruses from Eurasia.

These researchers found that the generalist subtype, HPAI H5, was driven largely by wild geese and swans that acted as a source for wild ducks, gulls, land birds, and domestic geese. Gulls were responsible for moving HPAI H5 more rapidly than any other host, a finding that may reflect their long-distance, pelagic movements and their immuno-naïve status against this subtype. Wild ducks, long viewed as primary hosts for spillover, occupied an optimal space for viral transmission, contributing to geographic expansion and rapid dispersal of HPAI H5. Evidence of interhemispheric dispersal via both the Pacific and Atlantic Rims was detected. Both neutral (geographic expansion) and non-neutral (antigenic selection) evolutionary processes were found to shape subtype evolution which manifested as unique geographic hotspots for each subtype at the global scale. This study reveals how a diversity of avian hosts contributes to viral spread and spillover.

None of this spread itself requires unusual climate or habitat changes – it is driven by long-habituated ‘normal’ bird migration and social behavior, albeit now accompanied by a highly pathogenic virus. The focus on ecological connections thus provides a more nuanced understanding than attributing spread only to random dispersal or the poultry trade. It becomes clear that biocontrol in one sector or region (e.g. European poultry farms) should not be considered as just a local endeavor, since infection dynamics in distant wild bird populations (African wetlands or Caspian colonies) can indirectly affect local risk via migratory traffic. For virologists and disease ecologists, these findings stress the importance of international surveillance and data-sharing. Genetic sequencing of AIV from wild birds in places like the Volga Delta, the Nile Delta, or West African reserves is invaluable for tracking viral evolution. It was through such sequencing that the “African PA gene” was detected in Caspian viruses and that the ultimate origin of Senegal’s outbreak was attributed to European migrants. Continuing the multidisciplinary, international research activity reflected in this paper will help predict new reassortants and potential phenotypic changes (e.g. mammalian adaptation, possibly dangerous to humans) as an early warning before they spread globally.

Knowledge gaps persist regarding AIV interactions with complex environmental components, comparative persistence of various strains under diverse natural conditions, and climate change impacts on environmental persistence and epidemiology (Breban et al., 2009; Williams et al., 2021; Zwarts et al., 2023). Development of improved methodologies for specific detection and quantification of infectious AIV in complex environmental matrices remains essential for enhanced surveillance and risk assessment capabilities.

Some waterbird species winter within the Caspian Basin itself, forming dense aggregations that can facilitate local pathogen amplification and persistence. Climate change-induced shifts in wintering patterns and locations of suitable waterbodies for foraging due to Caspian Sea rises and falls (Vilkov, 2014, 2016a, b, 2023) may alter these dynamics, potentially creating novel transmission opportunities. Milder winters are now enabling some typically migratory species to remain year-round in parts of the basin where they previously occurred only seasonally. These changes could create novel opportunities for pathogen maintenance during previously non-conducive periods. Extended AIV persistence in aquatic habitats has profound ecological and epidemiological implications. Environmental transmission through water predominates in temperate regions, contrasting with tropical ecosystems where direct respiratory transmission may be more significant. Wetland characteristics influence viral persistence potential, with small water bodies and high bird densities favoring transmission through reduced dilution effects and increased exposure opportunities. Seasonal persistence has significant variation, with highest detection rates during Palaearctic autumn migration and breeding periods, corresponding with immunologically naïve juvenile influx and increased aggregation densities.

Avian influenza viruses (AIV) can persist in environmental waters for varying durations depending on factors like temperature, salinity and virus strain (ECOPEACE Middle East, 2022). Studies show that some AIV subtypes can remain infectious for extended periods, potentially up to a year in some cases (Ramey et al., 2020, 2022; Poulson et al., 2024 – see also Stallknecht et al., 1990a, b, 2010; Keeler et al., 2012, 2013, 2014; Nielsen et al., 2013).

Recent outbreaks (e.g. the 2020s’ H5N1) show that the virus can now persist in wild populations all-year-round and reach places previously spared (even Antarctica) via migratory routes. This raises the stakes for monitoring Africa–Eurasia bird corridors, as viruses may exhibit bidirectional and multi-directional movement and can evolve along a migration route.

Encouragingly, international collaborative studies (such as those by the FAO, Wetlands International and a number of academic consortia) have filled many knowledge gaps since 2005, but challenges remain due to under-sampling in remote, inaccessible or under-funded areas and the inherent difficulty of tracking specific transmission events. In conclusion, the avian influenza landscape since 2005 has been truly intercontinental/global. Connectivity is both bidirectional and cyclical, forming a flyway network of viral circulation and exchange. For ecologists and virologists, this underscores the importance of an integrated international avian monitoring framework as conceived by One Health. Accurate forecasting of zoonotic risk requires models that couple climate projections with host-pathogen dynamics, habitat change and anthropogenic stressors. Such approaches are essential for designing adaptive conservation and public-health strategies in the climatically unstable, ecologically pivotal Caspian Basin (Krivenko and Vinogradov, 2008).

## 7 Conclusions

From 2005 to 2025, a picture emerges of a global avian influenza network linked by migratory waterbirds. The evidence gathered from field surveillance, phylogenetic analysis and ecological observation shows clear bidirectional virus flows between the Caspian Basin, Europe and Africa – albeit not equally in each direction. Generally, southward autumn migration has played a larger role in disseminating avian influenza, particularly highly pathogenic strains, from northern breeding areas (Europe/Caspian/Asia) to Africa. This has led to multiple incursions into Africa of H5 viruses (H5N1, H5N8, H5N6, etc.) since 2005. In parallel, low-pathogenic H9N2 remained established in poultry systems in Egypt and the Maghreb and has contributed gene segments to reassortant H5N1 in West Africa. On the other hand, the northward spring migration can return viruses or their descendants back to Europe and the Caspian, especially in the form of low-pathogenic strains or occasionally via HPAI in wild birds. The Caspian Basin, sitting at the intersection of routes, has been crucial both as a source (a stepping stone from Asia to Europe/Africa) and sink (receiving viruses from Europe/Africa).

The scientific literature to date paints a consistent picture: migratory waterbirds create a movable web of avian influenza exchange linking the Eurasian Palaearctic and Africa, with viruses moving south in the autumn and sometimes back north in the spring, evolving as they go. This bidirectional (indeed sometimes multidirectional) flow means that no region can be viewed in isolation. By drawing on two decades of studies, we can now appreciate the full scope of these dynamics, enabling better risk assessment and more informed public and animal health strategies across the northern and southern hemispheres. Only by accounting for the ancestry and trajectories of avian-borne viruses in wild bird hosts can the next virulent reassortant or epizootic wave be anticipated – whether it emerges in an Egyptian live-bird market, a Senegalese wetland or a remote Caspian nesting island.
