# Supplementary material for: Global dynamics of avian influenza: a twenty-year analysis of highly pathogenic viruses linking the Caspian Basin, Eurasia and Africa sectors (2005-2025)
Source: Front Cell Infect Microbiol. 2026 Jul 6;16:1795327. doi: 10.3389/fcimb.2026.1795327 (PMC13381235; doi:10.3389/fcimb.2026.1795327)
Supplement: Supplementary file 1 [file Table1.docx]

**SUPPLEMENTARY MATERIAL**

Table 1. Timeline and observed mortality during the West African seabird outbreak (Senegal and Gambia) and Species-specific mortality highlights.

| Period / date | Key observations / confirmations | Mortality counts reported |
| --- | --- | --- |
| Oct–Dec 2022 (sporadic phase) | Across seven sites, 17 sick birds and 14 mortalities recorded; first symptomatic cases at Tanji Bird Reserve and Sanyang (early Oct). Species found dead included West African crested terns, Caspian terns, Sandwich terns, and common terns at coastal locations (e.g., Gunjur, Kartong). Limited testing precluded confirmation. | 14 mortalities (plus 17 sick) |
| 15 Feb 2023 | Clinical signs consistent with HPAI documented (systematic surveillance phase). | — |
| 26 Feb 2023 | Two dead common terns discovered at Kartong Bird Observatory. | 2 dead common terns |
| 2 Apr 2023 | RT-PCR confirmation of HPAI H5N1 at Dakar laboratory from carcass swabs. | — |
| End Mar 2023 | Cumulative mortalities prior to peak. | 4,896 |
| Apr 2023 (peak) | Peak outbreak intensity during breeding season. | 13,632 adult deaths (April alone) |
| May 2023 | Declining but substantial mortality. | 4,086 |
| Jun 2023 | Further decline. | 1,943 |
| By Sep 2023 | Outbreak subsided. | — |
| Through Nov 2024 | Continued surveillance confirmed no additional incidents in either country. | — |

Species-specific mortality highlights reported for the West African seabird outbreak.

| Species / group | Deaths reported | Notes |
| --- | --- | --- |
| West African crested tern | >10,220 | ~40% of recorded deaths; ~4% of estimated breeding population (regional endemic) |
| Caspian tern | 4,686 | High mortality in breeding colonies |
| Grey-headed gull | 2,692 | Substantial losses |
| Sandwich tern | 2,394 | Losses coincided with March–May breeding season |

Table 2. Illustrative examples of bidirectional lineage/segment exchange and reassortment along the Africa/Southwest Asia-Caspian corridor (sources cited in text)

| Episode | Virus/hosts (summary) | Key genomic signatures | Inferred movement and outcomes |
| --- | --- | --- | --- |
| Volga Delta (northwest Caspian), 2020 | Egyptian-like H5N8 detected in the Volga Delta; wild hosts included black-headed gulls and waterfowl; reassortant genotypes (incl. EA-3) and an emergent H5N1 genotype detected later in Europe. | HA grouped with Egypt 2017-2019 H5N8; M gene traced to Egyptian-like virus; PB2/PA/NP clustered with Eurasian wild-bird viruses and with European H5N1 sequences from late 2020. | Suggested northward introduction from Egypt/SW Asia to Volga-Caspian early 2020; reassortment with Eurasian LPAI in the Caspian; by autumn 2020 dispersal west into Europe (including fall 2020 H5N1 detections in the Netherlands), seeding winter 2020-21 outbreaks in Europe and beyond to the Americas. |
| Maliy Zhemchuzhniy Island (north Caspian), May 2022 | H5N1 clade 2.3.4.4b associated with mass mortality in colonial waterbirds (notably Caspian terns) during the 2022 panzootic. | PA gene segment closely related to H5N1 strains identified in Africa (likely West African lineage 2020-2021); other segments (e.g., HA, NA, PB1, M) derived from European lineage viruses circulating in Europe/Middle East in winter 2021-22. | Evidence of south-to-north segment flow: African-derived PA segment incorporated into a Eurasian genetic background via reassortment somewhere along the flyway; ancestor hypothesized to overwinter in SW Asia (possibly Israel) before introduction to Caspian breeding colonies during spring migration. |
